# Priority of nutrition and exercise in depression management: triangulating mini-review of past and recent evidence with clinical practice guidelines

**DOI:** 10.1186/s41043-025-01138-0

**Published:** 2025-12-15

**Authors:** Shannon Rogers, Elizabeth Dean

**Affiliations:** 1 Rehabilitation Sciences Programs, Department of Occupational Science and Occupational Therapy, Department of Physical Therapy, Faculty of Medicine, University of British Columbia, Vancouver, Canada; 2 Department of Physical Therapy, Faculty of Medicine, University of British Columbia, Vancouver, BC Canada

**Keywords:** Anti-depressant, Anti-inflammatory, Diet, Nutrition, Physical activity; physical exercise

## Abstract

**Background:**

Chronic low-grade systemic inflammation (CLGSI) is implicated in depression and its amelioration. Pro-inflammatory nutrition and inactivity are associated with CLGSI. We triangulated the degree to which recent literature on anti-inflammatory nutrition and physical activity/physical exercise (PA/PE) corroborates the extant body of knowledge related to depression management and is reflected in the guidelines of leading mental health domains (national/international mental health associations/bodies).

**Methods:**

We used mini-review methodology. The search was narrowed to recent state-of-the-art literature (2024) in PubMed, on diet and exercise specifications in depression management. Then, we compared recommendations of aforementioned domains regarding diet and PA/PE in depression management.

**Results:**

Of 73 nutrition source studies, 50 (68%) focused on anti-inflammatory whole food, plant-based (WFPB) nutrition to manage depression; 4 (5%) on reducing consumption of animal-sourced foods; 16 (23%) focused on the effect of ultra-processed food (UPF) and its role in depression and its avoidance vis-à-vis its anti-depressant effects. Of the 55 PA/PE source studies, 49 (89%) focused on the effects of aerobic exercise; 14 (29%) described specific parameters to achieve an anti-depressant effect and 35 (71%) were non-specific. Twelve (22%) studies focused on resistance muscle training; 2 (17%) that reported specific training parameters and 10 (83%) that were non-specific. Nine domains were identified with established depression management guidelines: Australia/New Zealand; Canada; Europe (Belgium, Scotland, Spain); United Kingdom; United States; WFSBP and ASLM; and World Health Organization. Regarding nutrition, 5 (55%) domains recommended WFPB nutrition; 4 (44%) reduced animal-sourced foods; and 3 (33%), avoidance of UPF. With respect to sedentarism, 3 (33%) domains recommended reduced prolonged sitting. Eight domains (89%) mentioned aerobic exercise; 3 (33%) resistance training. Three domains mentioned aerobic exercise non-specifically; 5 (56%) made specific recommendations. Three domains mentioned resistance muscle training; 2 (22%) made non-specific recommendations and 1 (11%) made specific recommendations.

**Conclusions:**

Disparities that exist in leading depression management guidelines vis-à-vis inclusion of evidence-informed nutrition and PA/PE recommendations, warrant reconciliation. Evidence supporting anti-depressant WFPB nutrition and limiting pro-inflammatory animal-sourced food and UPF and supporting anti-inflammatory aerobic exercise and resistance training warrants being translated into national/international depression management guidelines as consistently as recommendations for pharmacotherapy and psychotherapy.

**Supplementary Information:**

The online version contains supplementary material available at 10.1186/s41043-025-01138-0.

## Background

Depression is the most common mental health condition affecting people worldwide and presents primarily or secondarily in combination with other pathologies [1, 2]. The Diagnostic and Statistical Manual of Mental Disorders describes multiple depression diagnoses including Depression; Depressive disorder due to another medical condition; Depressive disorder due to cerebrovascular disease; Disruptive mood dysregulation disorder; Major depressive disorder; Major depressive episodes and others [3]. The economic burden of these diseases is substantial and unsustainable [4, 5].

Contemporary management of disabling depression includes pharmacotherapy (medication), psychotherapy, and lifestyle modification [6]. Notwithstanding the importance of adequate sleep; manageable stress; and social connections; diet, and sedentarism and physical activity/physical exercise (PA/PE) independently and combined have been documented to contribute to depression bidirectionally in both positive and negative directions [7–16].

Chronic systemic low-grade systemic inflammation (CSLGI) is a common denominator of leading non-communicable diseases (NCDs) including depression [17–26]. Further, CSLGI is associated with the western lifestyle, i.e., consumption of the ubiquitous standard western diet and being sedentary and inactive [27–32]. The standard western diet has been well documented to be nutrient and fiber deficient (e.g., refined, grains, animal-sourced foods, and sugary foods and drinks), consistent with a pro-inflammatory state [33]. This ubiquitous eating pattern has been reported to be pro-oxidative and pro-inflammatory, the consequence of several characteristics that are highly deleterious to human health, e.g., high glycemic load; high fatty acid composition; poor macronutrient composition and quality; low micronutrient density; increased renal acid load and altered acid-base balance; reversal of the potassium-sodium ratio (to high sodium and low potassium); and pathologically low fiber content (characterized by low consumption of legumes, whole grains, vegetables and fruit) [34–37]. As a corollary, nutrient-dense, whole food, plant-based (WFPB) nutrition and regular moderate-intense PA/PE are anti-inflammatory [38–44], and have been reported to improve mood and reduce depression [19, 20, 38–49].

### Diet

Various dietary practices and their association with mental health have been studied. Over the past several decades, for example, the consumption of ultra-processed food (UPF) has become prevalent and increasingly a feature of the standard western diet [50]; its reduced consumption associated with improved mental health and reduced depressive symptoms [11, 51–55]. Other dietary regimens have been studied with respect to managing depression, often, if any, are associated with short-term remission including meat consumption that is pro-inflammatory [56–61], ketogenic diet [62–64], and low carbohydrate diet [65–67], and reduced sugar [68, 69].

### Physical activity/physical exercise

With respect to being physical inactive, sedentarism is a natural mental health depressant [6]. Thus, the avoidance of prolonged periods of sitting is protective from low mood states and depression. With respect to PA/PE and structured PE in the management of depression, a range of non-traditional alternative physical activities have been studied including tai chi [70, 71], dancing [72], gardening [73], mindfulness [74, 75], qi gong [76], mind body medicine [77], forest bathing [78], Pilates [79], and yoga [80].

### Diet and PA/PE combined

The anti-depressive efficacy of optimal nutrition and PA/PE with respect to not only reducing depressive symptoms but also reducing or eliminating the need for medication, is compelling, in both the interest of public health and reducing the economic burden. The potential of healthy nutrition to reduce depressive symptoms may reflect the phytonutrient density associated with fruit and vegetables [81–83]. Similarly, PA/PE appears equally effective in reducing depressive symptoms for individuals with co-morbidities as well as without, and with varying levels of baseline depression [18, 84]. PA/PE has been reported to be 1.5 times more effective at reducing mild-to-moderate symptoms of depression, psychological stress, and anxiety than medication or cognitive behavior therapy [84].

### Aim

The overarching aim of this study was to triangulate recent literature with the extant evidence on the roles of anti-inflammatory diet and PA/PE in the management of depression and the clinical practice guidelines for depression. Although based on observation, we predicted a discrepancy between the evidence and practice, the extent of that discrepancy was unclear.

## Methods

We conducted a mini-review of recent state-of-the-art evidence to provide a benchmark in the field with respect to specific anti-depressant, anti-inflammatory nutrition and PA/PE recommendations for depression management. First, we scoped the literature to identify diet and PA/PE parameters for potential modification of depression over a recent timeframe (2024) as a means of corroborating the state-of-the-art vis-à-vis the extant body of knowledge; second, to elucidate the degree to which clinical practice guidelines for the management of depression from recognized domains (i.e., national and international mental health bodies that have published formal clinical practice guidelines) include both dietary nutrition and PA/PE recommendations; and third, to establish the degree to which such recommendations reflect the evidence.

### Diet and PA/PE parameters for modification of depression

We used established procedures for conducting our triangulating mini-review that were relevant to our topic over one recent year, 2024, in order to corroborate the current state-of-the-art [85]. We chose one recent year given its contemporary relevance, and to establish that recent literature confirms and builds upon the body of extant evidence. We searched one representative, comprehensive database (PubMed) for related peer-reviewed literature. We included peer-reviewed studies using a range of experimental designs, evidence syntheses, epidemiological evidence, and reviews. Search terms included ‘depression’ or ‘depressive disorder’ or ‘depressive symptoms’ or ‘major depressive disorder’) AND (‘exercise’ or ‘physical fitness’ or ‘physical activity’) OR (‘diet’ or ‘nutrition’).

To simplify the associations between diet and PA/PE and depression, we chose to focus on primary depression as much as possible, i.e., without acknowledged co-morbidities. Articles that were excluded were those in the non-English literature (with one exception, Spain) and those related to secondary depression (e.g., associated with a chronic condition), substance abuse and addiction, non-human studies, isolated nutrient supplementation, study protocols, pediatrics, perinatal mothers, athletes, exercise-based gaming, living microbe intake, multifaceted approaches (those that included a range of interventions other than diet and exercise, e.g., psychological, group work, meditation), COVID-19, emotional eating, food preferences of those who are depressed, food insecurity, those articles that focused on both nutrition and PA/PE combined without differentiation of their effects, and topics for continuing medical education.

### Anti-depressant, anti-inflammatory diet and PA/PE recommendations in guidelines for depression management

With independent librarian assistance, we scoped established domains, i.e., organization and national/international bodies, that are dedicated to the needs and management of individuals with depression and published some form of clinical practice guidelines. We accessed the most recent versions of clinical practice guidelines and recommendations for the management of the needs of individuals with depression with special reference to dietary and PA/PE recommendations. We restricted our search to the following English-speaking industrialized countries: Australia and New Zealand, Canada, Europe (Belgium, Scotland, and Spain); United Kingdom; United States; and international collaborative guidelines including the World Health Organization. Recommendations from these nine domains associated with affluent regions, often have a ripple effect and influence guidelines and position papers published in low-middle income countries, lending further support for reviewing them.

Data extraction and tabulation were conducted by the investigators and cross-checked for accuracy. We extracted and enumerated the data based on categories of domain; whether diet and PA/PE recommendations were specified; and, if so, what these were specifically, verbatim.

## Results

**Fig. 1:**
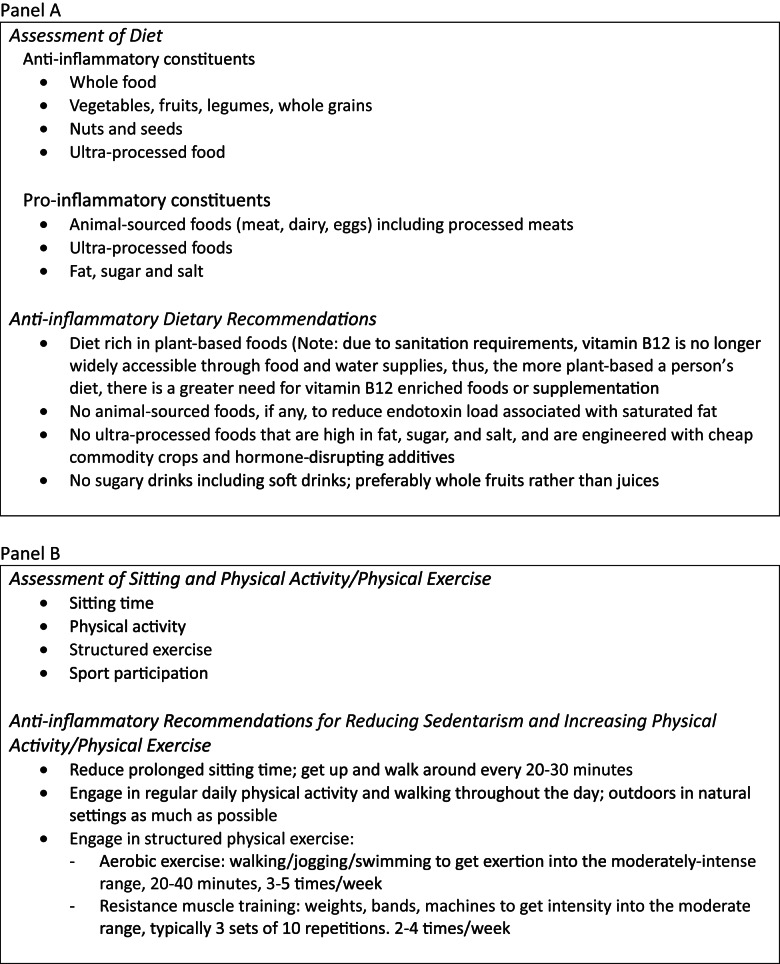
Evidence-based summary of diet (Panel **A**) and physical activity/physical exercise (Panel **B**) parameters and recommendations for prevention and modification of depression

Our search strategy yielded 73 sources studies related to nutrition and its effect on depression. These, their findings, and their implications vis-à-vis dietary recommendations are shown in Supplementary Table (1). The 55 source studies that were related to PA/PE, their findings, and their implications vis-à-vis PA/PE recommendations are shown in Supplementary Table (2). The An evidence-based summary of diet and physical activity/physical exercise parameters and recommendations for prevention and modification of depression appear in the Figure 1.

### Dietary parameters for modification of depression

Of 73 nutrition source studies (Supplementary Table 1), 50 (68%) focused on anti-inflammatory WFPB nutrition to manage depression; 4 (5%) on reducing consumption of animal-sourced foods; 16 (23%) focused on the effect of ultra-processed food (UPF) and its role in depression and its avoidance vis-à-vis its anti-depressant effects. Note, that some studies focused both on plant-based nutrition and avoidance of UPF.

### PA/PE parameters for modification of depression

Of the 55 PA/PE source studies (Supplementary Table 2), 49 (89%) focused on the effects of aerobic exercise; 14 (29%) described specific parameters to achieve an anti-depressant effect and 35 (71%) were non-specific. Twelve (22%) studies focused on resistance muscle training; 2 (17%) that reported specific training parameters and 10 (83%) that were non-specific. Note, that some studies focused both on aerobic and resistance muscle training.

### Diet and PA/PE guidelines for the management of depression published by established domains

Nine domains were identified with established guidelines: Australia/New Zealand; Canada; Europe (Belgium, Scotland, Spain); United Kingdom; United States; and World Federation of Societies for Biological Psychiatry (WFSBP) and Australasian Society of Lifestyle Medicine (ASLM); and World Health Organization. The diet and exercise guidelines for the management of depression from these established sources are summarized in Table 1.

**Table 1:** Diet and physical activity/physical exercise parameters for potential modification of depression recommended by nine established domains (associations and national and international health reports)

	Country/ Domain	Association and National/International Health Reports	Dietary recommendations and Co-morbidity implications	Exercise recommendations and Co-morbidity implications
1	Australia and New Zealand	Malhi et al. The 2020 Royal Australian and New Zealand College of Psychiatrists clinical practice guidelines for mood disorders. Aust N Z J Psychiatry. 2021;55(1):7–117	The evidence supporting the ‘Mediterranean diet’ characterised by high vegetable, fruit, fish and grain components, with low animal fat components, has increased The content of diet has an impact upon mood disorder symptoms, psychological well-being and overall health Ample amounts of fruits, vegetables (including potatoes), breads (particularly whole grain), nuts and seeds Fresh rather than processed food Fresh fruits as preferred desserts Dairy, poultry and fish in moderate quantities Red meats in small amounts Reduce saturated fats Olive oil (or other vegetable oil if preferred) as prime source of dietary fat Alcohol in modest quantities: maximum 1–2 standard drinks/day for males and 1 for females Methylfolates, probiotics and Omega-3 fatty acids may be helpful	Regular exercise is associated with improved quality of life, and antidepressant effects in depressive and bipolar disorders Exercise – particularly aerobic exercise – should be encouraged in all patients, not least because of its general health benefits Although the optimal amount and type of exercise is yet to be determined, we do know that health benefits require aerobic activity to be vigorous (enough to require concentrated effort) and regular (at least two to three times weekly) Resistance-based exercise is also beneficial in the management of depressive symptoms, and a combination of aerobic and resistance exercises is probably optimal Helping a patient build an exercise plan: Inform the patient that exercise is a helpful adjunctive treatment for depressive disorders because it improves brain function, offers pleasurable experiences, improves self-esteem, enhances sleep quality and increases the opportunities for interactions with other people Daily exercise is preferred over intermittent activity Aerobic (e.g., walking, running, cycling) and resistance exercise (e.g., using weights) are both effective for depressive disorders, and the combination may be better than either alone Some patients may find exercising with other people more motivating, but this is not universally the case Choose forms of exercise according to personal preference, remembering that previous preferences for exercise activities may help with this choice Ideally exercise should be pleasurable and require some effort, but not be exhausting or painful Documenting an exercise plan is helpful, along with regular reviews of progress and difficulties Some patients with depressive disorders struggle to find the ‘right exercise’ for them. Suggestions based upon their past activities and a review of options (e.g., start with a walk every day, perhaps with another person or pet) may be helpful Patients will benefit from building on their exercise plans to make these activities part of their daily lives; this may start as ‘treatment’ but could progress to becoming a ‘lifestyle’
2	Canada	Lam et al. Canadian Network for Mood and Anxiety Treatments (CANMAT) 2023 Update on Clinical Guidelines for Management of Major Depressive Disorder in Adults (CANMAT) 2023. Can J Psychiatry. 2024 Sep;69(9):641–687	Adjunctive healthy diet (varied diet with high content of fruit, vegetables, and fiber, and low content of saturated fat and carbohydrates)	Substantial evidence for the efficacy of exercise as a treatment for major depressive disorders adjunctive exercise is superior to no treatment and treatment as usual conditions in major depressive disorders, with medium to large effects Benefits in reducing depressive symptoms with the supervised aerobic activity of moderate to high intensity in adolescents and young adults, with low to moderate intensity exercise in midlife and older women, and with high-intensity interval training in adults First line: Supervised exercise (i.e., low to moderate intensity, for 30 to 40 min at a time, 3 to 4 times a wk, for a minimum of 9 wks) as a first-line monotherapy for mild depression and a second-line adjunctive treatment for moderate severity illness Supervised exercise (low to moderate intensity, for 30 to 40 min at a time, 3 to 4x/wk, for a minimum of 9 wks) for major depressive disorders of mild severity
3	Europe: Belgium	Medina et al. European clinical practice guidelines for depression in adults: Are they good enough? J Affect Disord. 2020 Feb 15;263:382–385. doi: 10.1016/j.jad.2019.12.005. Epub 2019 Dec 5. *Belgium* Karyotaki et al. (2014). *The long-term efficacy of psychotherapy*,* alone or in combination with antidepressants: The treatment of adult major depression* (KCE Report 230 C). Belgian Health Care Knowledge Centre (KCE)	None stated	None stated
4	Scotland	*Scotland*Scottish Intercollegiate Guidelines Network. (2010). *Non-pharmaceutical management of depression in adults* (SIGN Guideline No. 114). Healthcare Improvement Scotland	None stated	Structured exercise may be considered as a treatment option for patients with depressionExercise parameters are based on a limited number of studies including the following minimum requirements: three sessions/wk; 30–40 min duration; and a total energy expenditure of 17 kcal/kg/wkOther recommendations:5 sessions/wk, 30 min at a lower intensity with similar total energy expenditureRequired intensity of exercise correlated to energy expenditure of 70–80% of heart rate reserveCo-morbidities not addressed
5	SpainUnitedKingdom*(*see item 6)	*Spain*Working Group of the Clinical Practice Guideline on the Management of Depression in Adults. (2014). *Clinical practice guideline on the management of major depression in adults*. Ministry of Health, Social Services and Equality, Government of SpainUnited Kingdom*Guidelines from the United Kingdom in the Medina article were based on the 2010 NICE report; there are excluded here as these guidelines have been updated in 2022 and appear below	(From translation)None stated	(From translation)Limit sedentarismPhysical activity should be considered a part of treatment in adults with episodic depressionIn moderate depression, physical activity should be considered as a compliment to treatment either with antidepressant medications or structured psychological treatmentsA program of structured and supervised physical exercise for 30–60 min, 3 times/wk for ≥ 10–12 wksCo-morbidities not addressed
6	United Kingdom	National Institute of Health and Health and Clinical Excellence (NICE) Nice.org.uk/guidance/ng222 (2022)	Eating a healthy, balanced diet is an important part of maintaining good health, and can help you feel your best • eat at least 5 portions of a variety of fruit and vegetables every day (see 5 A Day) • base meals on higher fiber starchy foods like potatoes, bread, rice or pasta • have some dairy or dairy alternatives (such as soya drinks) • eat some beans, pulses, fish, eggs, meat and other protein (choose lean cuts of meat and skinless poultry whenever possible to cut down on fat; and less red and processed meat like bacon, ham and sausages) • choose unsaturated oils and spreads, and eat them in small amounts • drink plenty of fluids (at least 6 to 8 glasses a day) • Healthy Eating as a Vegan: this section is consistent with an anti-inflammatory diet	General Physical Activity Guidelines for Adults Aged 19 to 64 (also those 65 y and over) Provisos re other superimposed conditions • do strengthening activities that work all the major muscle groups (legs, hips, back, abdomen, chest, shoulders and arms) on at least 2 days/wk • do at least 150 min of moderate intensity activity a week or 75 min of vigorous intensity activity a week • spread exercise evenly over 4 to 5 days a week, or every day • reduce time spent sitting or lying down and break up long periods of not moving with some activity Group exercise with a trainer undertaking moderate intensity aerobic exercise Usually consists of ≥ 1 session/wk for 10 wks
7	United States	Guideline Development Panel for the Treatment of Depressive Disorders. Summary of the Clinical Practice Guideline for the Treatment of Depression Across Three Age Cohorts. American Psychological Association. Am Psychol. 2022;77(6):770–780	Nutrition was not a primary focus of the recommendations Guidelines acknowledge some evidence supporting dietary interventions, including the Mediterranean diet A diet rich in vegetables, fruits, fish, and grains, with low animal fats, appears beneficial for depressive disorders​ Olive oil is recommended as the primary source of dietary fat​ Red meat intake should be minimized Socioeconomic challenges must be considered when recommending dietary changes​ A dietitian or nutritionist may be required to support long-term adherence to dietary modifications​ **Co-morbidities** Diet and exercise interventions should be adapted for those with metabolic or cardiovascular conditions (e.g., obesity, hypertension) Monitoring weight, blood glucose, and lipid levels is essential in managing depressive patients with metabolic syndrome Depression may reduce motivation to engage in lifestyle changes, requiring structured support Family and community involvement in dietary and exercise programs can enhance adherence	Exercise and diet are beneficial for mild depressive symptoms Regular physical activity is strongly encouraged, with 150 min/wk of moderate exercise are recommended to help alleviate depressive symptoms Supervised exercise programs provide better adherence and outcomes than unsupervised regimens **General physical activity** Regular exercise is emphasized as a key intervention for depression management Daily exercise is preferable, and both aerobic (walking, running, cycling) and resistance training (weights) are effective​ Moderate and vigorous exercise (150–860 METs-min/wk) effectively reduces depression, anxiety, and stress​. Exercise enhances sleep quality, self-esteem, cognitive function, and neurotransmitter regulation​ **Group exercise programs for depression** Typically include moderate-intensity aerobic exercise for more than one session/wk for 10 wks 8 participants per group is typical Supervised programs (8–12 wks, 3–4 sessions/wk) improve functioning​ Exercise type includes walking, jogging, yoga, and strength training significantly reduce depression symptoms​
8	World Federation of Societies for Biological Psychiatry (WFSBP) and Australasian Society of Lifestyle Medicine (ASLM)	Marx et al. Clinical guidelines for the use of lifestyle-based mental health care in major depressive disorder: World Federation of Societies for Biological Psychiatry (WFSBP) and Australasian Society of Lifestyle Medicine (ASLM) taskforce. World J Biol Psychiatry. 2023 Jun;24(5):333–386	Encourage adherence to nutrient-dense, minimally processed dietary patterns, such as the Mediterranean diet Where required and available, refer to a trained dietician Increase consumption of fruits, vegetables, legumes, wholegrains, nuts, seeds, herbs and spices as tolerated Include a high consumption of foods rich in omega-3 polyunsaturated fatty acids and fiber Limit intake of ultra-processed foods and discretionary foods, and replace ultra-processed foods with minimally processed nutritious foods Consume red meat in moderation and opt for lean sources rather than processed and/or fatty cuts, considering the individual’s cultural-religious background Include extra virgin olive oil as the main source of cooking and added oil Consume the daily recommended water intake Avoid excessive alcohol consumption	Inquire about and encourage individuals to engage in modes of physical activity that they enjoy and at a frequency and intensity that they can sustain Discuss the use of supervised physical activity options such as group classes, use of personal trainers, and team sports as these may improve adherence in some individuals Engaging with an accredited exercise physiologist may be warranted especially where physical co-morbidities exist for individuals with major depressive disorders (e.g., heart conditions, COVID-19) to overcome barriers to participating in physical activity Pairing exercise with enjoyable activities can increase motivation e.g., listening to music, socializing, or exercising with a partner, watching television, or exercising in a pleasant environment Positive mental, physical, and social experiences acknowledged during physical activity (in real-time as opposed to retrospectively) can guide attention and increase motivation Encourage exercise routines that are feasible to implement most days rather than sporadically and that are effortful but not too difficult, exhausting, or painful. Examples may include commencing with initially low-moderate intensity exercise (e.g., walking/cycling short distances) rather than commencing with high intensity exercise routines (e.g., sprints, long distance running) Where feasible, work with individuals to gradually incorporate bouts of higher intensity exercise to gain maximal antidepressant benefits Provide examples of ways to reduce sedentary behavior as well as improving physical activity (e.g., digital apps and push notifications, reminders, standing/walking meetings, environmental modifications such as standing desks)
9	World Health Organization	mhGAP Intervention Guide Mental Health Gap Action Programme Version 2.0 for mental, neurological and substance use disorders in non-specialized health settings. 2016 https://www.who.int/publications/i/ item/9789241549790	Mention of assessing inappropriate diet along with other health-harming lifestyle behaviors and chronic disease ‘Try to eat regularly despite changes in appetite’	Mention of assessing physical inactivity along with other health-harming lifestyle behaviors and chronic disease ‘Try to be as physically active as possible’

With respect to nutrition (Table 2), 5 (55%) domains recommended WFPB nutrition; 4 (44%) reduced animal-sourced foods; and 3 (33%), avoidance of UPF. With respect to PA/PE (Table 2), 3 (33%) domains recommended reduced prolonged sitting (sedentarism). Eight domains (89%) mentioned aerobic exercise; 3 (33%) resistance training. Three domains mentioned aerobic exercise non-specifically; 5 (56%) made specific recommendations. Three domains mentioned resistance muscle training; 2 (22%) made non-specific recommendations and 1 (11%) made specific recommendations.

**Table 2:** Summary of the nine domainsthat included evidence-based depression management guidelines related to anti-inflammatory nutrition and physical activity/physical exercise

	Country/Domain	Whole food, plant based diet	Reduced animal-sourced foods	Avoid ultra-processed food	Reduced sedentarism	Aerobic	Resistance muscle training
1	Australia and New Zealand	√	√			√ Non-specific	√ Non-specific
2	Canada	√	√	√		√ Specific	
3	Europe: Belgium						
4	Europe: Scotland					√ Specific	
5	Europe: Spain				√	√ Specific	
6	United Kingdom	√	But does acknowledge vegan nutrition	√	√	√ Specific	√ Specific
7	United States	√	√			√ Specific	√ Non-specific
8	World federation of societies for biological psychiatry (WFSBP) and Australasian society of lifestyle Medicine (ASLM)	√	√	√	√	√ Non-specific	
9	World health organization					√ Non-specific	

## Discussion

Pharmacotherapy and psychotherapy remain the hallmark of contemporary depression treatment, either alone or with other treatment strategies. The findings of this mini-review however confirmed that the emphasis on anti-inflammatory nutrition and PA/PE in practice guidelines for depression, pales in comparison to the emphasis on pharmacotherapy and psychotherapy, which is not consistent with the weight of the evidence. Although nutrition and PA/PE interventions may have limitations and their implementation warrants being gauged to the needs of each individual, this is also true for the administration of pharmacotherapy and psychotherapy.

Consistent with a biomedical model, contemporary guidelines disproportionately weight psychotherapy and psychopharmacology [86]. As important as these interventions may have been shown to be, their emphasis tends to overshadow the contributions of the contemporary standard western diet and PA/PE practices that can contribute to and perpetuate depressive symptoms. Although the association of nutrition and mental health, for example, has been recognized for several decades giving rise to a psychiatric specialty of nutrition psychiatry, clinical practice guidelines for the management of psychiatric conditions have yet to fully integrate this body of knowledge into standard practice [87].

### Diet and PA/PE parameters for modification of depression

The optimal parameters for diet and PA/PE in the modification of depression are detailed below and are summarized in the Figure.

Diet.

With respect to diet, despite general agreement in the extant literature favoring WFPB nutrition in the management of depression, there were some anomalous findings in the recent literature that warrant explanation. For example, Apostolakopoulou and colleagues [8] concluded there were inconsistent reports regarding the anti-depressant effects of vegetarian diets. However, other than the confounded study by Saintila and colleagues that is described below [88], two source studies that defined vegetarian diet (e.g., consumption of fruits, vegetables, and whole grains) reported an inverse relationship with depression [89, 90] which supports earlier studies. Other anomalies, were two Asia studies that reported an inverse relationship between the consumption of fish and eggs, foods that are generally regarded as pro-inflammatory, and depressive symptoms [91, 92]. Compared with the standard western diet, the traditional Asian diets are more WFPB with lower fat, sugar and salt. One study of an American cohort reported that animal-sourced protein as well as anti-inflammatory vegetable protein and high-quality carbohydrate have a negative association with depression [93]. WFPB nutrition by definition is associated with high quality, unrefined carbohydrate content. Individual studies confirm such high-quality carbohydrate consumption is associated with low depression risk [18, 94] and low-quality, refined and highly processed carbohydrate is associated with depressive symptoms [95]. One study reported a higher caloric ratio of carbohydrate intake was associated with greater depression however the source of carbohydrate was not differentiated (complex or refined) [96], i.e., anti-inflammatory complex carbohydrates characteristic of WFPB nutrition, vs. pro-inflammatory simple carbohydrates characteristic of the standard western diet. Cheng and colleagues [97] reported that moderate carbohydrate (undefined) and higher protein consumption (plant or animal sources undifferentiated) were depression protective.

Two source articles failed to consider confounding sociodemographic variables, thus warrant particular mention to explain their exclusion from analysis. First, contrary to other evidence, Marche and colleagues reported that high protein consumption is associated with lower risk of depression [59]. In that study, the ratio of family income-to-poverty was lower (*p* < 0.001) for those study participants who were depressed. Individuals and families in lower socio-economic groups have been documented to eat less well and have nutritional deficiencies and are more likely to experience depression related to socioeconomic status and health issues [98, 99]. Second, Saintila and colleagues [88], reported that vegetarian diets are associated with increased symptoms of depression. Comparable to sociodemographic confounding variables in the study by Marche and colleagues (60), the vegetarians in the study by Saintila and colleagues were Peruvians with lower-socioeconomic status, compared with the non-vegetarians (*p* < 0.001) [88]. Those in low-socioeconomic groups have been documented to be more likely to consume nutrient-poor diets irrespective of whether they report being vegetarian or not. The United Nation’s Food and Agriculture Organization reported Peru has become the most food-insecure country in South America affecting over 50% of the population [100].

### Diet recommendations

Superimposed on the complexity of managing the needs of people who are depressed, is their reduced motivation to eat well including altered food preferences that are often processed and less healthy [101, 102], or to move and exercise [103, 104]. Thus, a detailed initial assessment is warranted to establish the patient’s current nutrition and exercise practices, as well as their histories, and readiness to consider such lifestyle changes in addition to their readiness to take medication or be referred for psychotherapy [105].

Compared with pharmacotherapy and psychotherapy, contemporary dietary and PA/PE recommendations are often categorized as adjunctive rather than among primary interventions when included in depression management approaches [86]. This is curious given that anti-inflammatory nutrition and PA/PE interventions may not only offset depressive symptoms but also can avert chronic NCDs that are often associated with depression [106]. These lifestyle interventions are also less invasive, have no adverse side-effects, are inexpensive, and are associated with greater assurance of life-long health overall.

Key findings from review of 2024 literature on diet and depression (Supplementary Table 1) corroborate extant literature described in the introduction. Inflammation contributes to depression and is also a consequence of depression [107]. Systemic inflammation influences neuroendocrine function and neurotransmitter metabolism, as well as synaptic efficiency. Proinflammatory signaling pathways also contribute to the pathogenesis of depression [29, 108]. High-fiber diets that are nutrient dense reduce inflammation by modulating the gut microbiome, in turn, influencing mood via the gut-brain axis [47, 107]. The gut-brain axis modulates intestinal inflammation [107]. Depressive symptoms have been associated with inflammatory compounds including sucrose, polyunsaturated fats and omega-6 linoleic acids, whereas anti-inflammatory compounds omega-3 (eicosapentaenoic acid) and vitamin C ameliorate depressive symptoms [109].

In one recent study of Australian women, Lee and colleagues concluded that depressive symptoms were reduced as long as the diet was high quality (defined as rich in plant foods) regardless of whether it was plant-based or not, and that a low-quality diet may increase depressive symptoms [110].

Overall mental health outcomes are improved with a diet rich in plant-based foods and less animal-sourced foods [111]. Diets that are higher in plant than animal content and fermented dairy products which encourage microbial diversity in the gut, can attenuate intestinal inflammation by modulating the gut microbiome [107]. One example is the EAT-Lancet diet primarily plant-based and emphasizes the intake of vegetables, fruits, whole grains and nuts [112]. It contains moderate amounts of seafood and poultry, and significantly limits red meat, and restricts added sugar and saturated fat. This dietary pattern limits animal-sourced foods, emphasizes higher plant content to better modulate the gut microbiome, thereby reduces depression and anxiety [112]. In one study, higher vegetable intake was linked to reduced depression, particularly in women, which is consistent with the literature. Participants reported fewer depressive symptoms with higher egg consumption, a finding that has not been independently corroborated [92].

Limiting meat consumption has been reported to reduce inflammation given animal protein is pro-inflammatory which does not support mental health [107]. However, some evidence suggests that protein sources including unprocessed red meat and poultry, are negatively correlated with the risk of depression as are the inclusion of dairy and nuts in the diet [93]. In one study, consuming chicken meat, fish, cereals, cheese as well as fruit, has been reported to be protective for depression and suggested that particularly in elderly populations, animal-sourced foods may reduce depression [58]. Overall, however, evidence strongly supports a plant-based diet such as the Mediterranean diet [112] for optimal mental health including reducing depression symptoms [88, 111]. Methodologically, these discrepancies warrant further investigation particularly in light of the well-documented health-harming effects of animal-sourced foods on human health [36]. Furthermore, endotoxins associated with meat produce symptoms resembling depression [113]. Bacterial toxins in the circulation that follows a meal of animal products results in inflammation and stiffened arteries, potentially exacerbated by the presence of saturated animal fat [114–116].

Much has been written about plant-based nutrition for maximal health and reduced disease risk including veganism [36]. Joy has providing evidence that eating animal-sourced foods is socially and culturally determined, rather than these foods being needed for maximal human nutrition [117]. Consistent with this (with the possible exception of the United Kingdom), is that 3 (33%) of the recommendations from the nine domains recommended a reduction of animal-sourced foods. Several leading global nutrition and dietetic associations have concurred globally that vegan nutrition is healthy across the life cycle including pregnant and lactating mothers [118]. However, largely due to sanitation requirements and contemporary agricultural practices, vitamin B12 is no longer widely accessible through food and water supplies. Thus, patients who are or become vegan, vitamin B12 enriched foods or supplementation is essential [36].

With respect to UPF, its consumption contributes to depression through inflammation-related mechanisms [119]. Sugar, for example, is a prime ingredient of processed foods. High-sugar diets are positively associated with depression in adults [44, 107]. Given diet’s impact on the gut microbiome, food choices can attenuate intestinal inflammation. High-sugar diets which include soft drinks and alcohol, disrupt the gut microbiota, leading to depression. Limiting the intake of sucrose and fat, basic ingredients of processed foods, may attenuate depressive symptoms by assisting the limbic system’s regulation of brain-derived neurotrophic factor [109]. Avoiding a pro-inflammatory diet including UPF such as junk food, fast food, processed meat, and alcoholic and soft drinks, lowers the risk of depression [49, 107].

Key findings from review of 2024 literature on PA/PE and depression (Supplementary Table 2) also corroborate extant literature described in the background of this review. Independently, sedentarism depresses mood and is associated with increased risk of depression in adults and older adult populations [120]. Regular PA/PE prevents depression onset regardless of age, sex or geographical region [121, 122]. PA/PE can be as effective as psychotherapy and antidepressant medication for treating depression even in those with transdiagnostic and subthreshold symptoms [18, 123]. Both aerobic exercise and resistance training are associated with significant therapeutic benefits when used as monotherapy or as adjuncts to medications and psychotherapy [124]. Some forms of physical exercise have been reported to be even more effective than selective serotonin reuptake inhibitors alone [18]. PA/PE can augment the effects of selective serotonin reuptake inhibitors; therefore, a combined exercise and medication approach may offer substantial benefits [18].

### PA/PE

The principles and parameters of PA/PE follow that are consistent with an anti-depressant effect.

### PA/PE recommendations

Despite the important of PA/PE, periods of prolonged sitting also need to be limited.

Sedentarism is an independent risk factor for multiple conditions including depression as was documented after the COVID-19 pandemic [125]. Thus, first and foremost, a case can be made for recommending reduced periods of prolonged sitting, to individuals who are depressed, as much as possible given the stage and severity of depression. Sitting time can be broken up every 20 to 30 min.

Even minimal amounts of PA/PE can decrease depressive symptoms [123]. Simply, increasing weekly energy expenditure by engaging in various physical activities including walking, jogging, yoga, and strength training can reduce levels of depressive symptoms [18, 80]. Combined aerobic and resistance training has been reported to reduce depression particularly in those with moderate symptoms [80]. Together, they showed moderate-to-large reductions in depressive symptoms and aerobic exercise was reported to be a particularly effective coping strategy [66].

Any intensity of PA/PE can be effective for reducing depression in a dose-dependent manner, however vigorous or intense exercise had the largest effect in the general adult population [18, 66]. For older adults, moderate intensity PA/PE appears to have the greatest effect on reducing depression until 81 years of age, after which its effect appears more limited [126, 127]. In frail and older adult populations, supervised individual or group interventions were particularly effective [127].

The predicted benefit of various PA/PE prescriptions depended upon co-morbidities, age and baseline levels of depression [18]. Sessions longer than 30 min were more effective in reducing depressive symptoms with weekly accumulation of only 150 min/week of moderate aerobic exercise [128]. Other studies described optimal energy expenditure to decrease symptoms in adults as being 600–1200 METs of light to moderate intensity [129]. However, in older adults, walking was effective in reducing depression in intensities over 250 METs/wk. Exceeding 1000 METs-mins/wk did not demonstrate substantial advantages in symptom reduction [80]. Various dose-response profiles for various physical activities were effective at 320 METs-min/wk and were optimized at 860 METs-min/wk [127]. Interventions ranging between 9 and 24 wks with a minimum duration of 8–12 wks, with supervised PE sessions occurring 385–4 times/wk were associated with the best outcomes [123].

In sum, PA/PE is an effective intervention in depression management and provides a potent alternative or adjunct to pharmacological approaches which are associated with side effects and limited long term efficacy [130]. There are also potential cost benefits to implementing group exercise interventions benefiting patients with depression and health systems [18].

### Diet and PA/PE recommendations in depression management guidelines

The contemporary recommendations for managing depression largely reflect the biomedical model with a focus on drugs and psychotherapy and less emphasis on lifestyle interventions known to impact depression including nutrition and PA/PE. Where there is attention to nutrition and PA/PE, recommendations are variable, and not consistently aligned with the evidence. Notably, however, the Canadian revised guidelines note that there is level one scientific evidence supporting exercise as a first-line lifestyle treatment for major depressive disorder of mild severity [131]. It includes clear and specific prescription parameters based on the evidence, i.e., frequency, intensity, time and type of PA/PE. The Canadian Network for Mood and Anxiety Treatments (CANMAT) also offers second-line lifestyle guidance for PA/PE as adjunctive therapy in moderate severity major depressive episodes. Adjunctive nutrition interventions including the Mediterranean diet and a diet with high fruit, vegetables and fiber, and low saturated fat and carbohydrates are considered third-line treatment recommendation. The inclusion of specific recommendations regarding diet and PA/PE sets this guideline apart from the others as the only clinical practice guideline which represents the science regarding the effects of diet and PA/PE on managing depression [131].

The literature supporting the benefit of WFPB nutrition for optimal mental health including depression as well as physical health (optimal physical health augments mental wellbeing) is established and unequivocal. Although all types of PA and PE appear to benefit mental health, there are dose-dependent benefits as described above. Compared with PA/PE recommendations, dietary recommendations are more uniform across individuals who may be or are depressed. PA/PE recommendations, on the other hand, need to consider type of depression, its presenting symptoms and impact on the patient, co-morbidities, individual preferences, age, and motivation. How best to tailor and target recommendations for diet and PA/PE and long-term adherence, for optimal mental health for a given individual, warrants being established with behavioral strategies including cognitive behavior therapy and motivational interviewing. Optimal nutrition and PA/PE prescription may help to avert the need for medication or, minimally, reduce its need.

Both the American College of Sports Medicine and the Canadian Society for Exercise Physiology recommended PE as an adjunctive treatment for individuals who have depressive disorders [132, 133]. Their specific recommendations are consistent with the literature. These two domains however were not included in those domains that we identified as providing clinical practice guidelines, as their focus was singularly PE. Having said that, the recommendations by the Canadian Society for Exercise Physiology also acknowledged diet and that it should be varied. Specific mention was made of the Mediterranean diet [133].

### Degree to which diet and PA/PE recommendations for depression reflect the current evidence base

Of the nine domains, the recommendations of the domains of Australia and New Zealand; United Kingdom; United States, and the WFSBP and ASLM were most aligned with the science vis-à-vis anti-inflammatory nutrition and PA/PE, with the latter domain being most aligned. These recommendations included WFPB nutrition and reduced animal-sourced foods. Only one domain, the WFSBP, specifically cautioned against consuming UPF, although some of the others inferred it.

### Strengths

Depression has been reported to have an associated inflammatory component. To our knowledge, this is the first examination of the congruence between the evidence related to the anti-inflammatory components of nutrition and PA/PE, and established clinical practice guidelines for depression management.

### Limitations

Although the science supporting WFPB nutrition and regular moderate-intense aerobic and resistance exercise being unequivocally associated with reduced oxidative stress and CLGSI contributing to depression, their inclusion into depression management regimens is but one aspect of comprehensive depression management, as substance abuse, poor sleep, social connections, and psychological stress need to be considered.

Further, similar to biomedical studies, the degree to which placebo plays a role in studies’ findings needs to be addressed. The issue of a placebo effect of exercise has been raised and warrants consideration in future study designs [134, 135]. The issue of placebo also may be a valid issue in relation to dietary practices as well.

Although some studies supported that the consumption of animal-sourced foods were inversely related to depression in some cohorts, this mini-review focused on the benefits of anti-inflammatory nutrition which has been documented to anti-depressant. As described, animal-sourced foods have been documented to be pro-inflammatory [136–138]. With respect to engaging in no exercise vs. anti-inflammatory exercise, no discrepancy has been reported in the literature. Typically, the focus of exercise-related studies is on the parameters of exercise that are associated with the maximal anti-depressant effects.

Our mini-review focused on general non-acute depression management. It is reasonable that the benefits of anti-inflammatory nutrition and PA/PE may benefit individuals with a range of depressive disorders to varying degrees. That said, comparable to the prescription of medication and psychotherapy, the timing of introducing nutrition and exercise interventions at a given stage in the patient’s course of depression, is based on a comprehensive assessment and ongoing evaluation. Comparable to other conditions, if depression becomes chronic, there is the potential that addressing lifestyle factors such as nutrition and PA/PE could prevent such episodes in the future and reduce the need for medication [139].

Finally, with respect to benchmarking the recent literature, we opted to use one representative established database. This was done for expediency to enable investigators to examine each study in sufficient detail to serve as a baseline for comparison with previous literature. That other databases might have yielded other findings is debatable. Also, in future studies and in the clinical practice guidelines that their findings inform, terms and their variants need to be clearly defined, e.g., ‘exercise’ and ‘diet’.

### Clinical and research implications and directions

Studies are needed to better prescribe anti-inflammatory diet and PA/PE to individuals with depression, e.g., readiness to introduce lifestyle changes. Individuals who are depressed have been reported to eat poorly and exercise less. Thus, assessing these individuals with respect to how they might most expediently and effectively shift their diets to being more plant-forward, and sit less and be aerobically active is paramount. Then, working with each individual to develop a unique program for each in improving the healthfulness of their lifestyle including anti-depressant, anti-inflammatory WFPB nutrition and PA/PE. Follow-up and provision of support are central to effecting change. Mixed methods research including qualitative research methods may shed more light on aspect to consider in both assessing individuals with depression on their lifestyles, and in prescribing individual lifestyle behavior change. Behavioral strategies including cognitive behavior therapy and motivational interview may have a role in empowering individuals with depression assume a pro-active role in addressing their symptoms.

Given the pervasive benefits of reduced sedentarism, and increased PA/PE on depressive symptoms, clinicians need to judiciously prescribe the appropriate dose of exercise based on the patient’s individual attributes and needs in conjunction with other indicated care. This integrated approach holds the promise of maximizing improvements in symptoms for patients with depressive symptoms. Some studies have shown that the benefits of PA/PE can be superior to medication, thus exploiting exercise is less costly and a more empowering alternative for patients [140, 141]. Future studies need to establish those who may benefit from this combination alone, i.e., nutrition and exercise, and for whom their benefit may be augmented with medication. Also, can this combination aid in the de-prescribing of medication in this patient cohort is provocative and warrants investigation. The relative effects of diet and exercise may vary from one individual to another, thus further study may elucidate this distinction. Future studies are also needed to elucidate the role of individual differences with respect to personal health autonomy and perceived control of their depressive symptoms and their capacity to influence them, e.g., differences impacted by gender, age, culture, and co-morbidity. Mixed-methods research is needed to investigate the barriers and facilitators of diet and PA/PE interventions for depression management from the perspectives of both the clinicians who recommend and support them, and those affected with depression who integrate these interventions into their symptom management.

Finally, despite evidence supporting an anti-inflammatory role underlying the benefit of an anti-oxidant, nutrient-dense, plant-rich diet and PA/PE to mental health, particularly depression, potential other mechanisms that warrant elucidation.

## Conclusion

Disparities that exist in leading depression management guidelines vis-à-vis inclusion of evidence-informed nutrition and PA/PE recommendations, warrant reconciliation. Evidence supporting anti-depressant WFPB nutrition and limiting pro-inflammatory animal-sourced food and UPF and supporting anti-inflammatory aerobic exercise and resistance training warrants being translated into national/international depression management guidelines as consistently as recommendations for pharmacotherapy and psychotherapy. We recommend that such integration be undertaken in the next iteration of the depression management guidelines published by the nine domains of interest, as well as new domains.

With attention to evidence-informed nutrition and PA/PE recommendations, not only may depression be prevented or offset in some individuals, but it may empower the person to exert control over their depressive symptoms and help reduce the need for medication. Maximizing personal control and agency is one means of augmenting mental health at a judicious time, such that depression is prevented or ameliorated. This relationship can be bidirectional so identifying the weighting of these factors in each individual presentation is imperative.

## Supplementary Information

Supplementary Material 1

## Data Availability

No datasets were generated or analysed during the current study.

## Abbreviations

ASLM: Australasian society of lifestyle medicine
CANMAT: Canadian network for mood and anxiety treatments
CLGSI: Chronic low-grade systemic inflammation
NCDs: Non-communicable diseases
PA: Physical activity
PE: Physical exercise
UPF: Ultra-processed food
WFPB: Whole food plant-based
WFSBP: World federation of societies for biological psychiatry

## References

[1] World Health Organization. Depression and other common mental disorders. Global Health Estimates. Geneva, Switzerland. 2017. Available from: https://iris.who.int/bitstream/handle/10665/254610/WHO-MSD-MER-2017.2-eng.pdf?sequence=1; accessed 29 August 2025.

[2] Gold SM, Köhler-Forsberg O, Moss-Morris R, Mehnert A, Miranda JJ, Bullinger M, et al. Comorbid depression in medical diseases. Nat Rev Dis Primers. 2020;6(1):69. 10.1038/s41572-020-0200-2. 32820163 10.1038/s41572-020-0200-2

[3] American Psychiatric Association. Diagnostic and statistical manual of mental disorders: DSM-5-TR [Internet]. Available from: https://www.psychiatry.org/patients-families/depression; accessed 29 August 2025.

[4] Greenberg PE, Fournier AA, Sisitsky T, Pike C, Kessler RC. The economic burden of adults with major depressive disorder in the united States (2005 and 2010). J Clin Psychiatry. 2015;76(2):155–62. 25742202 10.4088/JCP.14m09298

[5] World Health Organization. 2020. Depression. World Health Organization, Geneva. url: Available at: https://www.who.int/news-room/fact-sheets/detail/depression; accessed 29 August 2025.

[6] Harvard Medical School Special Health Report. Positive Psychology. Boston, MA: Harvard Health Publications; 2011.

[7] Firth J, Solmi M, Wootton RE, Vancampfort D, Schuch FB, Hoare E, et al. A meta-review of lifestyle psychiatry: the role of exercise, smoking, diet and sleep in the prevention and treatment of mental disorders. World Psychiatry. 2020;19(3):360–80. 32931092 10.1002/wps.20773 PMC7491615

[8] Apostolakopoulou XA, Petinaki E, Kapsoritakis AN, Bonotis K. A narrative review of the association between healthy dietary patterns and depression. Cureus. 2024;16(5):e60920. 10.7759/cureus.60920. 38910729 10.7759/cureus.60920 PMC11193411

[9] Bai Y, Guo S. Moderating effect of life’s essential 8 on the association of depression symptoms with all-cause and cardiovascular mortality. J Affect Disord. 2024;367:382–90. 10.1016/j.jad.2024.08.194. 39218311 10.1016/j.jad.2024.08.194

[10] ElBarazi A, Tikamdas R. Association between university student junk food consumption and mental health. Nutr Health. 2024;30(4):861–7. 36691314 10.1177/02601060231151480

[11] Ferreira NV, Gomes Gonçalves N, Khandpur N, Steele EM, Levy RB, Monteiro C et al. Higher ultraprocessed food consumption is associated with depression persistence and higher risk of depression incidence in the Brazilian longitudinal study of adult health. J Acad Nutr Diet. 2024;S2212-2672(24)00912-2, 630-40. 10.1016/j.jand.2024.10.012 39426518

[12] Sarris J, O’Neil A, Coulson CE, Schweitzer I, Berk M. Lifestyle medicine for depression. BMC Psychiatry. 2014;14:107. 10.1186/1471-244X-14-107. 24721040 10.1186/1471-244X-14-107 PMC3998225

[13] Ekinci GN, Sanlier N. The relationship between nutrition and depression in the life process: a mini-review. Exp Gerontol. 2023;172:112072. 10.1016/j.exger.2022.112072. 36565729 10.1016/j.exger.2022.112072

[14] Ünal G, Özenoğlu A. Association of mediterranean diet with sleep quality, depression, anxiety, stress, and body mass index in university students: a cross-sectional study. Nutr Health. 2024;2601060231207666. 10.1177/02601060231207666. 10.1177/02601060231207666 38280227

[15] Wang K, Zhao Y, Nie J, Xu H, Yu C, Wang S, Higher. HEI-2015 score is associated with reduced risk of depression: result from NHANES 2005–2016. Nutrients. 2021;13(2):348. 10.3390/nu13020348. 33503826 10.3390/nu13020348 PMC7911826

[16] Arocha Rodulfo JI. Sedentary lifestyle a disease from XXI century. Clin Investig Arterioscler. 2019;31(5):233–40. 31221536 10.1016/j.arteri.2019.04.004

[17] Dantzer R, O’Connor JC, Freund GG, Johnson RW, Kelley KW. From inflammation to sickness and depression: when the immune system subjugates the brain. Nat Rev Neurosci. 2008;9(1):46–56. 18073775 10.1038/nrn2297 PMC2919277

[18] Noetel M, Sanders T, Gallardo-Gómez D, Taylor P, Del Pozo Cruz B, van den Hoek D, et al. Effect of exercise for depression: systematic review and network meta-analysis of randomised controlled trials. BMJ. 2024;384:e075847. 10.1136/bmj-2023-075847. 38355154 10.1136/bmj-2023-075847 PMC10870815

[19] Yin W, Löf M, Chen R, Hultman CM, Fang F, Sandin S. Mediterranean diet and depression: a population-based cohort study. Int J Behav Nutr Phys Act. 2021;18(1):153. 10.1186/s12966-021-01227-3. 34838037 10.1186/s12966-021-01227-3 PMC8627099

[20] Zhao YL, Sun SY, Qin HC, Zhu YL, Luo ZW, Qian Y, et al. Research progress on the mechanism of exercise against depression. World J Psychiatry. 2024;14(11):1611–7. 39564183 10.5498/wjp.v14.i11.1611 PMC11572674

[21] Dowlati Y, Herrmann N, Swardfager W, Liu H, Sham L, Reim EK et al. A meta-analysis of cytokines in major depression.2010;67(5):446–57. 10.1016/j.biopsych.2009.09.033 20015486

[22] Osimo EF, Cardinal RN, Jones PB, Khandaker GM. Prevalence and correlates of low-grade systemic inflammation in adult psychiatric inpatients: an electronic health record-based study. Psychoneuroendocrinology. 2018;91:226–34. 29544672 10.1016/j.psyneuen.2018.02.031 PMC5910056

[23] Howren MB, Lamkin DM, Suls J. Associations of depression with C-reactive protein, IL-1, and IL-6: a meta-analysis. Psychosom Med. 2009;71(2):171–86. 19188531 10.1097/PSY.0b013e3181907c1b

[24] Abbas U, Hussain N, Tanveer M, Laghari RN, Ahmed I, Rajper AB. Frequency and predictors of depression and anxiety in chronic illnesses: a multi disease study across non-communicable and communicable diseases. PLoS One. 2025;20(5):e0323126. 10.1371/journal.pone.0323126. 40333937 10.1371/journal.pone.0323126 PMC12057975

[25] Hashmi AM, Butt Z, Umair M. Is depression an inflammatory condition? A review of available evidence. J Pak Med Assoc. 2013;63(7):899–906. 23901717

[26] Rosenblat JD, Cha DS, Mansur RB, McIntyre RS. Inflamed moods: a review of the interactions between inflammation and mood disorders. Prog Neuropsychopharmacol Biol Psychiatry. 2014;53:23–34. 24468642 10.1016/j.pnpbp.2014.01.013

[27] Berk M, Williams LJ, Jacka FN, O’Neil A, Pasco JA, Moylan S, et al. So depression is an inflammatory disease, but where does the inflammation come from? BMC Med. 2013;11:200. 10.1186/1741-7015-11-200. 24228900 10.1186/1741-7015-11-200 PMC3846682

[28] Milaneschi Y, Kappelmann N, Ye Z, Lamers F, Moser S, Jones PB, et al. Association of inflammation with depression and anxiety: evidence for symptom-specificity and potential causality from UK biobank and NESDA cohorts. Mol Psychiatry. 2021;26(12):7393–402. 34135474 10.1038/s41380-021-01188-w PMC8873022

[29] Burini RC, Anderson E, Durstine JL, Carson JA. Inflammation, physical activity, and chronic disease: an evolutionary perspective. Sports Med Health Sci. 2020;2(1):1–6. 35783338 10.1016/j.smhs.2020.03.004 PMC9219305

[30] Francis H, Stevenson R. The longer-term impacts of Western diet on human cognition and the brain. Appetite. 2013;63:119–28. 23291218 10.1016/j.appet.2012.12.018

[31] Wang F, Sun M, Wang X, Wu Z, Guo R, Yang Y, et al. The mediating role of dietary inflammatory index on the association between eating breakfast and depression: based on NHANES 2007–2018. J Affect Disord. 2024;348:1–7. 10.1016/j.jad.2023.12.015. 38070746 10.1016/j.jad.2023.12.015

[32] Sahabudhee A, Rao CR, Chandrasekaran B, Pedersen SJ. Dose-response effects of periodic physical activity breaks on the chronic inflammatory risk associated with sedentary behavior in high- and upper-middle income countries: a systematic review and meta-analysis. Diabetes Metab Syndr. 2023;17(3):102730. 10.1016/j.dsx.2023.102730. 36863092 10.1016/j.dsx.2023.102730

[33] Lucas M, Chocano-Bedoya P, Schulze MB, Mirzaei F, O’Reilly ÉJ, Okereke OI, et al. Inflammatory dietary pattern and risk of depression among women. Brain Behav Immun. 2014;36:46–53. 10.1016/j.bbi.2013.09.014. 24095894 10.1016/j.bbi.2013.09.014 PMC3947176

[34] Cordain L, Eaton SB, Sebastian A, Mann N, Lindeberg S, Watkins BA, et al. Origins and evolution of the Western diet: health implications for the 21st century. Am J Clin Nutr. 2005;81(2):341–54. 15699220 10.1093/ajcn.81.2.341

[35] García-Montero C, Fraile-Martínez O, Gómez-Lahoz AM, Pekarek L, Castellanos AJ, Noguerales-Fraguas F, et al. Nutritional components in Western diet versus Mediterranean diet at the gut microbiota-immune system interplay: implications for health and disease. Nutrients. 2021;13(2):699. 10.3390/nu13020699. 33671569 10.3390/nu13020699 PMC7927055

[36] Dean E, Xu J, Jones AY, Vongsirinavarat M, Lomi C, Kumar P, et al. An unbiased, sustainable, evidence-informed universal food guide: a timely template for national food guides. Nutr J. 2024;23(1):126. 10.1186/s12937-024-01018-z. 39425106 10.1186/s12937-024-01018-z PMC11487974

[37] Storz MA, Ronco AL, Hannibal L. Observational and clinical evidence that plant-based nutrition reduces dietary acid load. J Nutr Sci. 2022;11:e93. 10.1017/jns.2022.93. 36405093 10.1017/jns.2022.93 PMC9641522

[38] WHO European Office for the Prevention and Control of Noncommunicable Diseases. Plant-based diets and their impact on health, sustainability and the environment: a review of the evidence. Available at: https://www.who.int/europe/publications/i/item/WHO-EURO-2021-4007-43766-61591; accessed 28 Aug 2025.

[39] Hepsomali P, Kagami-Katsuyama H, Coxon C, Honma N, Kinoshita K, Hattori H, et al. Dietary inflammation, sleep and mental health in the United Kingdom and Japan: a comparative cross-sectional study. Nutr Bull. 2024;49(3):396–407. 39001567 10.1111/nbu.12695

[40] Fayyazi E, Mohammadi E, Aghamohammadi V. Association between major dietary patterns and mental health problems among college students. J Educ Health Promot. 2024;13:440. 10.4103/jehp.jehp_1405_23. 39811839 10.4103/jehp.jehp_1405_23 PMC11731339

[41] Mahajan H, Lieber J, Carson Mallinson PA, Bhogadi S, Banjara SK, Kinra S, et al. Higher dietary inflammation is associated with a higher burden of Multimorbidity of cardiometabolic and mental health disorders in an urbanizing community of Southern india: a cross-sectional analysis for the APCAPS cohort. Hum Nutr Metab. 2024;36:200254. 10.1016/j.hnm.2024.200254. 10.1016/j.hnm.2024.200254 PMC11052728 38828398

[42] Sangsefidi ZS, Hosseinzadeh M, Lorzadeh E, Sangsefidi ZS, Salehi-Abarghouei A, Mirzaei M. The association between dietary antioxidant quality score and psychological disorders among Iranian adults: a population-based study. Nutr Neurosci. 2024;27(1):12–9. 36495152 10.1080/1028415X.2022.2153430

[43] Varaee H, Mirzaei M, Salehi-Abargouei A, Ahmadi N, Hosseinzadeh M. Evaluation of lifestyle and dietary inflammatory score and their relationship with the odds of depression, stress, and anxiety in adults living in Yazd, iran: based on YaHS and TAMYZ cohort study. J Affect Disord. 2024;347:486–91. 38000473 10.1016/j.jad.2023.11.069

[44] Zhang L, Sun H, Liu Z, Yang J, Liu Y. Association between dietary sugar intake and depression in US adults: a cross-sectional study using data from the National Health and Nutrition Examination Survey 2011–2018. BMC Psychiatry. 2024;24(1):110. 10.1186/s12888-024-05531-7. 38326834 10.1186/s12888-024-05531-7 PMC10851576

[45] Haghighatdoost F, Mahdavi A, Mohammadifard N, Hassannejad R, Najafi F, Farshidi H, et al. The relationship between a plant-based diet and mental health: evidence from a cross-sectional multicentric community trial (LIPOKAP study). PLoS ONE. 2023;18(5):e0284446. 10.1371/journal.pone.0284446. 37256886 10.1371/journal.pone.0284446 PMC10231825

[46] Lassale C, Batty GD, Baghdadli A, Jacka F, Sánchez-Villegas A, Kivimäki M, et al. Healthy dietary indices and risk of depressive outcomes: a systematic review and meta-analysis of observational studies. Mol Psychiatry. 2019;24(7):965–86. 30254236 10.1038/s41380-018-0237-8 PMC6755986

[47] Lin J, Gao Y, Shen Q, Li J, Zhou Z, Shen L. Dietary flavonoid intake is associated with a lower risk of depressive symptoms in US adults: data from NHANES 2007–2008, 2009–2010, and 2017–2018. J Affect Disord. 2024;345:293–9. 37890540 10.1016/j.jad.2023.10.128

[48] Wu H, Gu Y, Meng G, Wu H, Zhang S, Wang X, et al. Quality of plant-based diet and the risk of dementia and depression among middle-aged and older population. Age Ageing. 2023;52(5):afad070. 10.1093/ageing/afad070. 37247402 10.1093/ageing/afad070

[49] Selvaraj R, Selvamani TY, Zahra A, Malla J, Dhanoa RK, Venugopal S, et al. Association between dietary habits and depression: a systematic review. Cureus. 2022;14(12):e32359. 10.7759/cureus.32359. 36632273 10.7759/cureus.32359 PMC9828042

[50] Marino M, Puppo F, Del Bo’ C, Vinelli V, Riso P, Porrini M, et al. A systematic review of worldwide consumption of ultra-processed foods: findings and criticisms. Nutrients. 2021;13(8):2778. 10.3390/nu13082778. 34444936 10.3390/nu13082778 PMC8398521

[51] Arshad H, Recchia D, Head J, Holton K, Norton J, Kivimaki M, et al. Adherence to MIND diet and risk of recurrent depressive symptoms: prospective Whitehall II cohort study. Nutrients. 2024;16(23):4062. 10.3390/nu16234062. 39683455 10.3390/nu16234062 PMC11643367

[52] de Farias Xavier DE, de Moraes RCS, Viana TAF, Pereira JKG, da Costa PCT, Duarte DB, et al. Food consumption according to the NOVA food classification and its relationship with symptoms of depression, anxiety, and stress in women. Nutrients. 2024;16(21):3734. 10.3390/nu16213734. 39519567 10.3390/nu16213734 PMC11547796

[53] Ghernati L, Tamim H, Chokor FAZ, Taktouk M, Assi B, Nasreddine L, et al. Processed and ultra-processed foods are associated with depression and anxiety symptoms in a cross-sectional sample of urban Lebanese adults. Nutr Res. 2025;133:172–89. 39764859 10.1016/j.nutres.2024.11.011

[54] Mengist B, Lotfaliany M, Pasco JA, Agustini B, Berk M, Forbes M, et al. The risk associated with ultra-processed food intake on depressive symptoms and mental health in older adults: a target trial emulation. BMC Med. 2025;23(1):172. 10.1186/s12916-025-04002-4. 40128798 10.1186/s12916-025-04002-4 PMC11934811

[55] Contreras-Rodríguez O, Reales-Moreno M, Fernández-Barrès S, Cimpean A, Arnoriaga-Rodríguez M, Puig J, et al. Consumption of ultra-processed foods is associated with depression, mesocorticolimbic volume, and inflammation. J Affect Disord. 2023;335:340–8. 37207947 10.1016/j.jad.2023.05.009

[56] Dobersek U, Bender M, Etienne A, Fernandez Gil GE, Hostetter C. Meat consumption and positive mental health: a scoping review. Prev Med Rep. 2023;37:102556. 10.1016/j.pmedr.2023.102556. 38186660 10.1016/j.pmedr.2023.102556 PMC10770626

[57] Forootani B, Sasanfar B, Salehi-Abargouei A, Mirzaei M. The association between plant and animal protein intake with depression, anxiety, and stress. Nutr Neurosci. 2025;28(3):370–83. 38980695 10.1080/1028415X.2024.2372194

[58] Marche C, Poulain M, Nieddu A, Errigo A, Dore MP, Pes GM. Is a plant-based diet effective to maintain a good psycho-affective status in old age? Results of a survey of a long-lived population from Sardinia. Nutr Neurosci. 2024;27(4):382–91. 37023016 10.1080/1028415X.2023.2198115

[59] Marche C, Baourakis G, Fakotakis E, Nieddu A, Errigo A, Pes GM. The impact of nutrition on psycho-affective status in an older Cretan population: a cross-sectional study. Eur J Nutr. 2024;63(6):2199–207. 38744756 10.1007/s00394-024-03395-x

[60] Wang Y, Uffelman C, Hill E, Anderson N, Reed J, Olson M, et al. The effects of red meat intake on inflammation biomarkers in humans: a systematic review and meta-analysis of randomized controlled trials. Curr Dev Nutr. 2022;6(Suppl 1):994. 10.1093/cdn/nzac068.023.

[61] Kostovcikova K, Coufal S, Galanova N, Fajstova A, Hudcovic T, Kostovcik M, et al. Diet rich in animal protein promotes pro-inflammatory macrophage response and exacerbates colitis in mice. Front Immunol. 2019;10:919. 10.3389/fimmu.2019.00919. 31105710 10.3389/fimmu.2019.00919 PMC6497971

[62] Calabrese L, Frase R, Ghaloo M. Complete remission of depression and anxiety using a ketogenic diet: case series. Front Nutr. 2024;11:1396685. 10.3389/fnut.2024.1396685. 38887496 10.3389/fnut.2024.1396685 PMC11182043

[63] Chrysafi M, Jacovides C, Papadopoulou SK, Psara E, Vorvolakos T, Antonopoulou M, et al. The potential effects of the ketogenic diet in the prevention and co-treatment of stress, anxiety, depression, schizophrenia, and bipolar disorder: from the basic research to the clinical practice. Nutrients. 2024;16(11):1546. 10.3390/nu16111546. 38892480 10.3390/nu16111546 PMC11174630

[64] Laurent N, Bellamy EL, Hristova D, Houston A. Ketogenic diets in clinical psychology: examining the evidence and implications for practice. Front Psychol. 2024;15:1468894. 10.3389/fpsyg.2024.1468894. 39391844 10.3389/fpsyg.2024.1468894 PMC11464436

[65] Arsalandeh F, Shemirani F, Nazari MA, Mirmiran P, Golzarand M. Effect of low-carbohydrate diets on quality of life, mental health, and speed of memory in adults: a systematic review and meta-analysis of randomised controlled trials. Int J Food Sci Nutr. 2025;76(1):4–19. 39617964 10.1080/09637486.2024.2430006

[66] Chen C, Du S, Shao Q, Fu X, Jin L, Zhou S, et al. The effects of aerobic exercise for depression: an umbrella review of systematic reviews and meta-analyses. J Bodyw Mov Ther. 2024;40:2161–72. 39593579 10.1016/j.jbmt.2024.10.068

[67] Walaszek M, Kachlik Z, Cubała WJ. Low-carbohydrate diet as a nutritional intervention in major depressive disorder: focus on relapse prevention. Nutr Neurosci. 2024;27(10):1185–98. 38245881 10.1080/1028415X.2024.2303218

[68] Barma MD, Purohit BM, Priya H, Malhotra S, Bhadauria US, Duggal R. Sweet misery: association of sugar consumption with anxiety and depression - A systematic review. Obes Rev. 2025 ; Jul 26:e70003. 10.1111/obr.70003 40715023

[69] Jacques A, Chaaya N, Beecher K, Ali SA, Belmer A, Bartlett S. The impact of sugar consumption on stress driven, emotional and addictive behaviors. Neurosci Biobehav Rev. 2019;103:178–99. 31125634 10.1016/j.neubiorev.2019.05.021

[70] Kraft J, Waibl PJ, Meissner K. Stress reduction through taiji: a systematic review and meta-analysis. BMC Complement Med Ther. 2024;24(1):210. 10.1186/s12906-024-04493-3. 38831412 10.1186/s12906-024-04493-3 PMC11149313

[71] Kuang X, Dong Y, Song L, Dong L, Chao G, Zhang X, et al. The effects of different types of Tai Chi exercise on anxiety and depression in older adults: a systematic review and network meta-analysis. Front Public Health. 2024;11:1295342. 10.3389/fpubh.2023.1295342. 38259770 10.3389/fpubh.2023.1295342 PMC10800705

[72] Prudente TP, Mezaiko E, Silveira EA, Nogueira TE. Effect of dancing interventions on depression and anxiety symptoms in older adults: a systematic review and meta-analysis. Behav Sci. 2024;14(1):43. 10.3390/bs14010043. 38247695 10.3390/bs14010043 PMC10813489

[73] Wood CJ, Barton J, Wicks CL. Effectiveness of social and therapeutic horticulture for reducing symptoms of depression and anxiety: a systematic review and meta-analysis. Front Psychiatry. 2025;15:1507354. 10.3389/fpsyt.2024.1507354. 39917376 10.3389/fpsyt.2024.1507354 PMC11799672

[74] Kardel KR, Iversen PO, Kaaya AN, Muhoozi G, Veierød MB, Wangen KR, et al. A pragmatic randomized trial to examine the effect of combining healthy diet with mindfulness cognitive therapy to reduce depressive symptoms among university students in a low-resource setting: protocol for the nutrimind project. BMC Psychiatry. 2024;24(1):610. 10.1186/s12888-024-06056-9. 39261786 10.1186/s12888-024-06056-9 PMC11391632

[75] Huang H, Huang S, Chen S, Gao X, Cai J, Feng Y, et al. Interventions for psychiatric disorders among university students: an umbrella review of systematic reviews and meta-analyses. Int J Clin Health Psychol. 2024;24(1):100431. 10.1016/j.ijchp.2023.100431. 38287943 10.1016/j.ijchp.2023.100431 PMC10823073

[76] Dong J, Wang D, Li H, Ni H. Effects of different Chinese traditional exercises on sleep quality and mental health of adults: systematic review and meta-analysis. Sleep Breath. 2024;28(1):29–39. 37474686 10.1007/s11325-023-02881-6

[77] Kucukosmanoglu HS, Cramer H, Tavakoly R, Moosburner A, Bilc MI. Mind-body medicine in the treatment of depression: a narrative review of efficacy, safety and mechanisms. Curr Psychiatry Rep. 2024;26(12):729–40. 39424743 10.1007/s11920-024-01548-7 PMC11706891

[78] Piva G, Caruso L, Gómez AC, Calzolari M, Visintin EP, Davoli P, et al. Effects of forest walking on physical and mental health in elderly populations: a systematic review. Rev Environ Health. 2022;39(1):121–36. 36239186 10.1515/reveh-2022-0093

[79] Ju M, Zhang Z, Tao X, Lin Y, Gao L, Yu W. The impact of Pilates exercise for depression symptoms in female patients: a systematic review and meta-analysis. Medicine. 2023;102(41):e35419. 10.1097/MD.0000000000035419. 37832060 10.1097/MD.0000000000035419 PMC10578749

[80] Tian S, Liang Z, Tian M, Qiu F, Yu Y, Mou H, et al. Comparative efficacy of various exercise types and doses for depression in older adults: a systematic review of paired, network and dose-response meta-analyses. Age Ageing. 2024;53(10):afae211. 10.1093/ageing/afae211. 39348911 10.1093/ageing/afae211

[81] Lim SY, Kim EJ, Kim A, Lee HJ, Choi HJ, Yang SJ. Nutritional factors affecting mental health. Clin Nutr Res. 2016;5(3):143–52. 27482518 10.7762/cnr.2016.5.3.143 PMC4967717

[82] Saghafian F, Malmir H, Saneei P, Milajerdi A, Larijani B, Esmaillzadeh A. Fruit and vegetable consumption and risk of depression: accumulative evidence from an updated systematic review and meta-analysis of epidemiological studies. Br J Nutr. 2018;119(10):1087–101. 29759102 10.1017/S0007114518000697

[83] Zhou YF, Song XY, Pan XF, Feng L, Luo N, Yuan JM, et al. Association between combined lifestyle factors and healthy ageing in Chinese adults: the Singapore Chinese health study. The Journals of Gerontology: Series A. 2021;76(10):1796–805. 10.1093/gerona/glab033 PMC8436980 33522576

[84] Singh B, Olds T, Curtis R, Dumuid D, Virgara R, Watson A, et al. Effectiveness of physical activity interventions for improving depression, anxiety and distress: an overview of systematic reviews. Br J Sports Med. 2023;57(18):1203–9. 36796860 10.1136/bjsports-2022-106195 PMC10579187

[85] Griffiths P. Evidence informing practice: introducing the mini-review. Br J Community Nurs. 2002;7(1):38–9. 11823729 10.12968/bjcn.2002.7.1.9435

[86] Guideline Development Panel for the Treatment of Depressive Disorders. Summary of the Clinical Practice Guideline for the Treatment of Depression Across Three Age Cohorts. American Psychological Association. Am Psychol. 2022;77(6):770 – 80. 10.1037/amp0000904 34843274

[87] American Psychiatry Association. How to Boost Mental Health Through Better Nutrition. Available at: https://www.psychiatry.org/news-room/apa-blogs/mental-health-through-better-nutrition; accessed 29 August 2025.

[88] Saintila J, Carranza-Cubas SP, Serpa-Barrientos A, Carranza Esteban RF, Cunza-Aranzábal DF, Calizaya-Milla YE. Depression, anxiety, emotional eating, and body mass index among self-reported vegetarians and non-vegetarians: a cross-sectional study in Peruvian adults. Nutrients. 2024;16(11):1663. 38892596 10.3390/nu16111663 PMC11174459

[89] Bojang KP, Manchana V. Impact of vegetarianism on cognition and neuropsychological status among urban community-dwelling adults in Telangana, South India: a cross-sectional study. Eur J Nutr. 2024;63(4):1089–101. 10.1007/s00394-024-03328-8. 38305863 10.1007/s00394-024-03328-8

[90] Gomes-da-Costa S, Fernandéz-Pérez I, Borras R, Lopez N, Rivas Y, Ruiz V, et al. Is a vegetarian diet beneficial for bipolar disorder? Relationship between dietary patterns, exercise and pharmacological treatments with metabolic syndrome and course of disease in bipolar disorder. Acta Psychiatr Scand. 2024;150(4):209–22. 10.1111/acps.13733. 38994686 10.1111/acps.13733

[91] Kim E, Je Y. Fish consumption and depression in Korean population: the Korea National health and nutrition examination survey, 2013–2021. J Affect Disord. 2024;359:255. 38782264 10.1016/j.jad.2024.05.103

[92] Kitabayashi M, Umetsu S, Suzuki M, Konta T. Relationship between food group-specific intake and depression among local government employees in Japan. BMC Nutr. 2024;10(1):21. 10.1186/s40795-024-00830-4. 38291535 10.1186/s40795-024-00830-4 PMC10826071

[93] Dang X, Yang R, Jing Q, Niu Y, Li H, Zhang J, et al. Association between high or low-quality carbohydrate with depressive symptoms and socioeconomic-dietary factors model based on XGboost algorithm: from NHANES 2007–2018. J Affect Disord. 2024;351:507–17. 38307135 10.1016/j.jad.2024.01.220

[94] Song X, He K, Xu T, Tian Z, Zhang J, He Y, et al. Association of macronutrient consumption quality, food source and timing with depression among US adults: a cross-sectional study. J Affect Disord. 2024;351:641–8. 38309482 10.1016/j.jad.2024.01.252

[95] Naghshi N, Tehrani AN, Rabiei S, Behrouz V, Yari Z. Association between different dietary carbohydrate and risk of depression, anxiety, and stress among female adolescents. Int J Prev Med. 2024;15:71. 10.4103/ijpvm.ijpvm_291_23. 39742124 10.4103/ijpvm.ijpvm_291_23 PMC11687680

[96] Tan Y, Yu S, Cao Y, Guo X, Tang W, Zou X. Higher caloric ratio of carbohydrate intake associated with increased risk of depression: a cross-sectional analysis of NHANES data from 2005 to 2020. J Affect Disord. 2024;366:59–65. 39209272 10.1016/j.jad.2024.08.179

[97] Cheng Z, Fu F, Lian Y, Zhan Z, Zhang W. Low-carbohydrate-diet score, dietary macronutrient intake, and depression among adults in the united States. J Affect Disord. 2024;352:125–32. 38367707 10.1016/j.jad.2024.02.054

[98] Siddiqui F, Salam RA, Lassi ZS, Das JK. The intertwined relationship between malnutrition and poverty. Front Public Health. 2020;8:453. 10.3389/fpubh.2020.00453. 32984245 10.3389/fpubh.2020.00453 PMC7485412

[99] Ridley M, Rao G, Schilbach F, Patel V. Poverty, depression, and anxiety: causal evidence and mechanisms. Science. 2020;370(6522):eaay0214. 10.1126/science.aay0214. 33303583 10.1126/science.aay0214

[100] United Nations, Food and Agriculture Organization. Peru’s food crisis grows amid soaring prices and poverty [Internet]. New York: United Nations. 2022 [cited 2025 Apr 17]. Available from: https://news.un.org/en/story/2022/11/1130737; accessed 29 August 2025.

[101] Sedgi FM, Hejazi J, Derakhshi R, Baghdadi G, Zarmakhi M, Hamidi M, et al. Investigation of the relationship between food preferences and depression symptoms among undergraduate medical students: a cross-sectional study. Front Nutr. 2025;12:1519726. 10.3389/fnut.2025.1519726. 40129668 10.3389/fnut.2025.1519726 PMC11930683

[102] Thurn L, Schulz C, Borgmann D, Klaus J, Ellinger S, Walter M, et al. Altered food liking in depression is driven by macronutrient composition. Psychol Med. 2025;55:e20. 39905823 10.1017/S0033291724003581 PMC12017361

[103] Codella R, Chirico A. Physical inactivity and depression: the gloomy dual with rising costs in a large-scale emergency. Int J Environ Res Public Health. 2023;20(2):1603. 10.3390/ijerph20021603. 36674363 10.3390/ijerph20021603 PMC9862474

[104] Elfrey MK, Ziegelstein RC. The inactivity trap. Gen Hosp Psychiatry. 2009;31(4):303–5. 19555788 10.1016/j.genhosppsych.2009.05.001 PMC2752478

[105] DiClemente CC, Prochaska JO. Toward a comprehensive, transtheoretical model of change—stages of change and addictive behaviors. In: Miller WR, Heather N, editors. Treating addictive behaviors. Volume 2. New York, NY: Springer; 1998 , pp.1-24.

[106] National Institute of Mental Health. Understanding the link between chronic disease and depression [Internet]. Available from: https://www.nimh.nih.gov/health/publications/chronic-illness-mental-health; accessed 29 August 2025.

[107] van Zonneveld SM, van den Oever EJ, Haarman BCM, Grandjean EL, Nuninga JO, van de Rest O, et al. An anti-Inflammatory diet and its potential benefit for individuals with mental disorders and neurodegenerative diseases. A narrative review. Nutrients. 2024;16(16):2646. 10.3390/nu16162646. 39203783 10.3390/nu16162646 PMC11357610

[108] Wang Q, Ou Z, Chen J, Li L, Chen Y, Ye D. Association between vegetable intake and major depressive disorder: results from National Health and Nutrition Examination Survey 2005–2018 and bidirectional two-sample Mendelian randomisation. Public Health Nutr. 2024;27(1):e220. 10.1017/S1368980024001691. 39445502 10.1017/S1368980024001691 PMC11604328

[109] Serefko A, Jach ME, Pietraszuk M, Świąder M, Świąder K, Szopa A. Omega-3 polyunsaturated fatty acids in depression. Int J Mol Sci. 2024;25(16):8675. 10.3390/ijms25168675. 39201362 10.3390/ijms25168675 PMC11354246

[110] Lee M, Ball L, Hill S, Crowe TC, Walsh H, Cosgrove T, et al. Omnivore, vegan and vegetarian diet quality associations with depressive symptoms: a comparative cross-sectional analysis of the Australian longitudinal study on women’s health. J Affect Disord. 2025;370:18–25. 39477074 10.1016/j.jad.2024.10.119

[111] Ross FC, Mayer DE, Gupta A, Gill CIR, Del Rio D, Cryan JF, et al. Existing and future strategies to manipulate the gut microbiota with diet as a potential adjuvant treatment for psychiatric disorders. Biol Psychiatry. 2024;95(4):348–60. 37918459 10.1016/j.biopsych.2023.10.018

[112] Lu X, Wu L, Shao L, Fan Y, Pei Y, Lu X, et al. Adherence to the EAT-lancet diet and incident depression and anxiety. Nat Commun. 2024;15(1):5599. 38961069 10.1038/s41467-024-49653-8 PMC11222463

[113] DellaGioia N, Hannestad J. A critical review of human endotoxin administration as an experimental paradigm of depression. Neurosci Biobehav Rev. 2010;34(1):130–43. 19666048 10.1016/j.neubiorev.2009.07.014 PMC2795398

[114] Erridge C. The capacity of foodstuffs to induce innate immune activation of human monocytes *in vitro* is dependent on food content of stimulants of Toll-like receptors 2 and 4. Br J Nutr. 2011;105(1):15–23. 20849668 10.1017/S0007114510003004

[115] Erridge C, Attina T, Spickett CM, Webb DJ. A high-fat meal induces low-grade endotoxemia: evidence of a novel mechanism of postprandial inflammation. Am J Clin Nutr. 2007;86(5):1286–92. 17991637 10.1093/ajcn/86.5.1286

[116] Ghanim H, Abuaysheh S, Sia CL, Korzeniewski K, Chaudhuri A, Fernandez-Real JM, et al. Increase in plasma endotoxin concentrations and the expression of Toll-like receptors and suppressor of cytokine signaling-3 in mononuclear cells after a high-fat, high-carbohydrate meal: implications for insulin resistance. Diabetes Care. 2009;32(12):2281–7. 19755625 10.2337/dc09-0979 PMC2782991

[117] Joy M. Why we love dogs, eat pigs, and wear cows [monograph]. Newburyport, MA: Conari; 2010.

[118] Albert Schweitzer Foundation. Vegan: healthy across all stages of life cycle [Internet]. Available from: https://albertschweitzerfoundation.org/news/vegan-diet-healthy-across-all-stages-of-life-cycle; accessed 29 August 2025.

[119] Contreras-Rodriguez O, Reales-Moreno M, Fernández-Barrès S, Cimpean A, Arnoriaga-Rodríguez M, Puig J, et al. Consumption of ultra-processed foods is associated with depression, mesocorticolimbic volume, and inflammation. J Affect Disord. 2023;335:340–8. 37207947 10.1016/j.jad.2023.05.009

[120] Jiang Y, Zhang M, Cui J. The relationship between sedentary behavior and depression in older adults: a systematic review and meta-analysis. J Affect Disord. 2024;362:723–30. 39032707 10.1016/j.jad.2024.07.097

[121] Alderman BL, Perdue HM, Sarwani AH. Exercise for the prevention and treatment of depression. Curr Top Behav Neurosci. 2024;67:157–75. 39042248 10.1007/7854_2024_496

[122] Rahmati M, Lee S, Yon DK, Lee SW, Udeh R, McEvoy M, et al. Physical activity and prevention of mental health complications: an umbrella review. Neurosci Biobehav Rev. 2024;160:105641. 10.1016/j.neubiorev.2024.105641. 38527637 10.1016/j.neubiorev.2024.105641

[123] García-Estela A, Angarita-Osorio N, Holzhausen MC, Mora-Salgueiro J, Pérez V, Duarte E, et al. Evaluating the effect of exercise-based interventions on functioning in people with transdiagnostic depressive symptoms: a systematic review of randomised controlled trials. J Affect Disord. 2024;351:231–42. 38278328 10.1016/j.jad.2024.01.191

[124] Rossi FE, Dos Santos GG, Rossi PAQ, Stubbs B, Barreto Schuch F, Neves LM. Strength training has antidepressant effects in people with depression or depressive symptoms but no other severe diseases: a systematic review with meta-analysis. Psychiatry Res. 2024;334:115805. 10.1016/j.psychres.2024.115805. 38428290 10.1016/j.psychres.2024.115805

[125] Meyer JD, O’Connor J, McDowell CP, Lansing JE, Brower CS, Herring MP. High sitting time is a behavioral risk factor for blunted improvement in depression across 8 weeks of the COVID-19 pandemic in April–May 2020. Front Psychiatry. 2021;12:741433. 10.3389/fpsyt.2021.741433. 34658975 10.3389/fpsyt.2021.741433 PMC8519400

[126] Kong LN, Lyu Q, Liu DX, Hu P. Effects of exercise interventions on physical, psychological and social outcomes in frail older adults: an overview of systematic reviews. J Clin Nurs. 2024. 10.1111/jocn.17214. 38716880 10.1111/jocn.17214

[127] Tang L, Zhang L, Liu Y, Li Y, Yang L, Zou M, et al. Optimal dose and type of exercise to improve depressive symptoms in older adults: a systematic review and network meta-analysis. BMC Geriatr. 2024;24(1):505. 10.1186/s12877-024-05118-7. 38849780 10.1186/s12877-024-05118-7 PMC11157862

[128] Smith PJ, Merwin RM. The role of exercise in management of mental health disorders: an integrative review. Annu Rev Med. 2021;72:45–62. 33256493 10.1146/annurev-med-060619-022943 PMC8020774

[129] Li X, He S, Liu T, Zhang X, Zhu W, Wang C, et al. Impact of exercise type, duration, and intensity on depressive symptoms in older adults: a systematic review and meta-analysis. Front Psychol. 2024;15:1484172. 10.3389/fpsyg.2024.1484172. 39346508 10.3389/fpsyg.2024.1484172 PMC11427357

[130] Zhao S, Tang Y, Li Y, Shen H, Liu A. Associations between life’s essential 8 and depression among US adults. Psychiatry Res. 2024;338:115986. 10.1016/j.psychres.2024.115986. 38850892 10.1016/j.psychres.2024.115986

[131] Lam RW, Kennedy SH, Adams C, Bahji A, Beaulieu S, Bhat V, et al. Canadian network for mood and anxiety treatments (CANMAT) 2023 update on clinical guidelines for management of major depressive disorder in adults: Réseau canadien pour les traitements de l’humeur et de l’anxiété (CANMAT) 2023: mise à Jour des Lignes directrices cliniques pour La prise En charge du trouble dépressif Majeur Chez les adultes. Can J Psychiatry. 2024;69(9):641–87. 38711351 10.1177/07067437241245384 PMC11351064

[132] Ozemek C, Bonikowske A, Christle J, Gallo P. Chapter 11: brain health and brain-Related disorders. ACSM’s guidelines for exercise testing and prescription. 12th ed. Ozemek C, Bonikowske A, Christle J, Gallo P (Eds). Philadelphia (PA): Wolters Kluwer; 2025. pp. 378–424.

[133] Canadian Society for Exercise Physiology. CSEP Exercise & Depression Specialization™ – Preparation Module 2: Treatment Approaches [Internet]. Ottawa (ON): Canadian Society for Exercise Physiology; 2025 [cited 2025 May 20]. Available from: https://store.csep.ca/products/csep-exercise-depression-specialization-preparation-module-2-treatment-approaches; accessed 29 August 2025.

[134] Blumenthal JA, Babyak MA, Doraiswamy PM, Watkins L, Hoffman BM, Barbour KA, et al. Exercise and pharmacotherapy in the treatment of major depressive disorder. Psychosom Med. 2007;69(7):587–96. 17846259 10.1097/PSY.0b013e318148c19a PMC2702700

[135] Raglin JS, Lindheimer JB. The placebo effect in exercise and mental health research. Curr Top Behav Neurosci. 2024;67:381 – 94. 10.1007/7854_2024_506. PMID: 39042249. 10.1007/7854_2024_506 39042249

[136] Barbaresko J, Koch M, Schulze MB, Nöthlings U. Dietary pattern analysis and biomarkers of low-grade inflammation: a systematic literature review. Nutr Rev. 2013;71(8):511–27. 23865797 10.1111/nure.12035

[137] Fighting Chronic Inflammation. Harvard medical School. Special health report. Boston, MA: Harvard Health Publishing; 2024.

[138] Sivasubramanian BP, Dave M, Panchal V, Saifa-Bonsu J, Konka S, Noei F, et al. Comprehensive review of red meat consumption and the risk of cancer. Cureus. 2023;15(9):e45324. 10.7759/cureus.45324. 37849565 10.7759/cureus.45324 PMC10577092

[139] Ornish D, Ornish A. UnDo it! How simple lifestyle changes can reverse most chronic diseases (monograph). New York (NY): Ballantyne Books; 2019.

[140] Belliveau R, Horton S, Hereford C, Ridpath L, Foster R, Boothe E. Pro-inflammatory diet and depressive symptoms in the healthcare setting. BMC Psychiatry. 2022;22(1):125. 10.1186/s12888-022-03771-z. Erratum in: BMC Psychiatry. 2022;22(1):838. 35172770 10.1186/s12888-022-03771-z PMC8851832

[141] Blumenthal JA, Babyak MA, Doraiswamy PM, Watkins L, Hoffman BM, Barbour KA, et al. Exercise and pharmacotherapy in the treatment of major depressive disorder. Psychosom Med. 2007;69(7):587–96. 17846259 10.1097/PSY.0b013e318148c19a PMC2702700

