# Supplementary material for: Priority of nutrition and exercise in depression management: triangulating mini-review of past and recent evidence with clinical practice guidelines
Source: J Health Popul Nutr. 2025 Dec 15;44:421. doi: 10.1186/s41043-025-01138-0 (PMC12706896; doi:10.1186/s41043-025-01138-0)
Supplement: Supplementary file 1 — Supplementary Material 1 [file 41043_2025_1138_MOESM1_ESM.docx]

**Supplementary Table 1** Summary of recent 2024 literature on effects of diet related to the potential modification of depression

| No. | Study Title | Dietary Recommendations |
| --- | --- | --- |
| 1 | Alnabulsi et al. Adherence to the Mediterranean diet in Saudi Arabia and its association with socioeconomic status and depression. Medicina (Kaunas). 2024 Apr 17;60(4):642. | Supports an association between following the MedDiet and decreased incidence of severe depression, supporting a role for the MedDiet in enhancing mental health |
| 2 | Apostolakopoulou et al. A Narrative review of the association between healthy dietary patterns and depression. Cureus. 2024 May 23;16(5):e60920. doi: 10.7759/cureus.60920. | Healthy eating patterns (high in vegetables, fruits, whole grains, nuts, seeds, and fish, low in processed foods), i.e., associated with lower Dietary Inflammatory Index, are related to a reduction in the risk of depression  Most robust findings are related to MedDiet and the DASH diet  Regarding the vegetarian diet, there are inconsistent reports  Eating patterns of people with depression have poorer nutritional quality, with lower fruit and vegetable intake  Strengthens the argument that nutritional interventions should be incorporated as an important "pillar" in the multifactorial treatment of patients with depression |
| 3 | Arshad et al. Association between ultra-processed foods and recurrence of depressive symptoms: the Whitehall II cohort study. Nutr Neurosci. 2024;27(1):42-54. | Confirms the prospective ultra-processed food (UPF) intake-depression outcomes association in a Western non-Mediterranean country where greater UPF consumption is evident compared to Mediterranean countries  Lends support to strategies to limit the consumption of UPF |
| 4 | Arslan et al. Determination of the relationship between dietary inflammatory index and depression status in female students. Nutr Health. 2024;30(4):707-713. | High intake of ultra-processed foods is associated with increased odds of recurrent depressive symptoms and contributes to the overall diet quality-depressive symptoms association |
| 5 | Ataei Kachouei et al. Relationship of the Prime Diet Quality Score (PDQS) and Healthy Eating Index (HEI-2015) with depression and anxiety: a cross-sectional study. BMC Public Health. 2024 Oct 22;24(1):2919. doi: 10.1186/s12889-024-20369-0. | Both PDQS and HEI-2015 (both weight fruit, vegetable, whole grains, low fat consumption) were associated with a reduced risk of depression and anxiety; the PDQS demonstrated a stronger inverse association with these risks compared to the HEI-2015  The PDQS which has greater anti-inflammatory constituents, could be a more beneficial dietary pattern for preventing depression and anxiety compared to HEI-2015 |
| 6 | Bahrami et al. The association between soft drinks consumption and risk of mental disorders among Iranian adults: The LIPOKAP study. J Affect Disord. 2024 Oct 15;363:8-14. | Positive association between soft drinks, sugar-sweetened beverages, and artificial juice and depression |
| 7 | Bai et al. Association of Life's Essential 8 with depression among adults: A cross-sectional study of NHANES. J Investig Med. 2024 Oct;72(7):737-746. | Higher overall LE8 scores and higher scores for each component (healthy diet that is high in vegetables, fruits, whole grains, nuts, seeds; physical activity; nicotine exposure; sleep duration; body mass index; blood lipids; blood glucose; and blood pressure) were associated with lower odds of depression  LE8 score might be a useful tool for both cardiologists and psychiatrists in screening for and monitoring mental as well as physical health  Primary care physicians also could better tailor care and interventions to address mental as well as physical health needs, with nutrition counselling |
| 8 | Bayes et al. The use of diet for preventing and treating depression in young men: current evidence and existing challenges. Br J Nutr. 2024 Jan 28;131(2):214-218. | Diet was based on the Australian Guide to Healthy Eating, with additional Mediterranean-style diet components resulted in significantly lower self-reported depression symptoms  Together, these findings suggest that following a Mediterranean Diet has the potential to have a wide impact on young men with depression, influencing many aspects of their health and well-being  The Mediterranean Diet was well tolerated in both of these studies with no reported side effects |
| 9 | Bojang et al. Impact of vegetarianism on cognition and neuropsychological status among urban community-dwelling adults in Telangana, South India: a cross-sectional study. Eur J Nutr. 2024;63(4):1089-1101. | Consumption of fruits, vegetables, and whole grains, common components of vegetarian diets, linked to a reduced likelihood of experiencing depression and anxiety  Vegetarianism exerted a positive influence on the cognitive and neuropsychological status of the investigated population  Mini mental state examination scores were inversely correlated with depression, anxiety, and stress for vegetarians |
| 10 | Cabrera-Suárez et al. Mediterranean diet-based intervention to improve depressive symptoms: analysis of the PREDIDEP randomized trial. Nutr Neurosci. 2024;27(9):951-961. | Positive effect of a Mediterranean diet intervention on depression in short-medium term (for two years) |
| 11 | Campisi et al. A personalised nutrition intervention for adolescent depression: a mixed-methods feasibility pilot study. Br J Nutr. 2024 Oct 29:1-13. | A 1-week personalised menu plan was co-created based on the principles of the Mediterranean diet (i.e., higher intake of fruit, vegetables, grains, legumes and lean protein and lower intake of red meats and processed foods) for use over the following 2 weeks  Benefits with respect to depression symptoms and family food dynamics |
| 12 | Canhada et al. Associations of ultra-processed food intake with the incidence of cardiometabolic and mental health outcomes go beyond specific subgroups-The Brazilian Longitudinal Study of Adult Health. Nutrients. 2024 Dec 12;16(24):4291. doi: 10.3390/nu16244291. | UPFs overall and their subgroups predicted future cardiometabolic and mental health outcomes  The pattern of individual UPF subgroup associations varied across outcomes, and the aggregate of subgroups not individually predicting risk also predicted large gains in overall and central adiposity and the incidence of mental health disorders  Findings justify avoidance of overall UPF intake in health promotion and disease prevention |
| 13 | Chen et al. Association of Life's Essential 8 with incident cardiovascular disease among individuals with depression: A prospective study. Can J Cardiol. 2024;40(12):2640-2648. | Optimal adherence to Lifestyle Essential 8 was associated with lower burden of cardiovascular disease in those with depression  Adopting a comprehensive lifestyle intervention might help further reduce cardiovascular disease burden in those with mental disorders |
| 14 | Cheng et al. Low-carbohydrate-diet score, dietary macronutrient intake, and depression among adults in the United States. J Affect Disord. 2024;352:125-132. | Inverse association observed between low carbohydrate diet score and risk of depression mainly resulted from the inverse association between protein intake and risk of depression  Both excessive and low carbohydrate intake were reported to increase risk of depression  Moderate carbohydrate intake and high protein intake were correlated with a lower risk of depression  Inverse association between protein intake and the risk of depression |
| 15 | Choi et al. Healthy and unhealthy dietary patterns of depressive symptoms in middle-aged women. Nutrients. 2024 Mar 8;16(6):776. | Healthy dietary patterns that involve consuming a variety of foods such as whole grains, legumes, nuts, vegetables and fruits, fish, seafood, and milk and dairy products reduce the risk of depressive symptoms  A diet high in fiber appears to reduce depressive symptoms by regulating neurotransmitters and creating a beneficial microbial environment  Unhealthy dietary patterns involving the consumption of foods such as cakes, cookies, chocolate, carbonated drinks, coffee, and meat increased the risk of depressive symptoms in Korean middle-aged women  A diet that includes a variety of fiber-rich foods prevented and managed depressive symptoms in middle-aged women  To prevent depression, educate the public to increase their intake of fruits, vegetables, and whole grains, and to promote policies to increase the consumption of these foods |
| 16 | Conner et al. Effect of moderate red meat intake compared with plant-based meat alternative on psychological well-being: A 10-wk cluster randomized intervention in healthy young adults. Curr Dev Nutr. 2024 Nov 16;9(1):104507. doi: 10.1016/j.cdnut.2024.104507. | No effect on the psychological measures and limited change to physiological status when comparing a balanced diet containing either red meat or plant-based meat alternatives in healthy young adults |
| 17 | Dai et al. Ultra-processed foods and human health: An umbrella review and updated meta-analyses of observational evidence. Clin Nutr. 2024 Jun;43(6):1386-1394. | High UPF consumption is associated with an increased risk of a variety of chronic diseases and mental health disorders  At present, not a single study reported an association between UPF intake and a beneficial health outcome  Dietary patterns with low consumption of UPFs may render broad public health benefits |
| 18 | Dang et al. Association between high or low-quality carbohydrate with depressive symptoms and socioeconomic-dietary factors model based on XGboost algorithm: From NHANES 2007-2018. J Affect Disord. 2024 Apr 15;351:507-517. | Negative association between high-quality carbohydrates and depressive symptoms and a positive association between low-quality carbohydrates and depressive symptoms  Carbohydrate quality is associated with depressive symptoms, and machine learning models that combine diet with socioeconomic factors can be a tool for predicting depression severity. |
| 19 | Darabi et al. The association between lifelines diet score (LLDS) with depression and quality of life in Iranian adolescent girls. Nutr J. 2024 Feb 15;23(1):19. doi: 10.1186/s12937-024-00913-9. | Based on the 2015 Dutch Dietary Guidelines and underlying literature, nine food groups with positive (vegetables, fruit, whole grain products, legumes and nuts, fish, oils and soft margarines, unsweetened dairy, coffee and tea) and three food groups with negative health effects (red and processed meat, butter and hard margarines and sugar sweetened beverages) constituted the lifelines diet  Inverse relationship between LLDS with risk of depression and poor QoL |
| 20 | de Farias Xavier et al. Food consumption according to the NOVA Food Classification and its relationship with symptoms of depression, anxiety, and stress in women. Nutrients. 2024 Oct 31;16(21):3734. | Women with depressive symptoms had a lower intake of unprocessed and minimally processed foods (fruits, greens and other vegetables), i.e., negatively correlated with symptoms of depression, anxiety, and stress compared with controls |
| 21 | Deng et al. Association between dietary flavonoid intake and depressive symptoms: A cross-sectional research. Gen Hosp Psychiatry. 2024 Jan-Feb;86:75-84. | Dietary flavonoid intake (includes onions, kale, broccoli, apples, leaks, berries, celery, grapes, cabbage) was associated with the decreased probability of depression symptoms in U.S. adults, among which flavones, flavanones, and anthocyanidins may occupy the predominant roles |
| 22 | Diaz-Amaya et al. Food security and diet quality, not vitamin D status are significantly associated with depression: Results from NHANES 2015-2018. J Affect Disord. 2024 Feb 15;347:150-155. doi: 10.1016/j.jad.2023.11.071. Epub 2023 Nov 22. | Food security and diet quality were significantly associated with depression  Highlights how evaluating food security and diet quality (assessed with the Healthy Eating Index which leans toward being anti-inflammatory) are critical in research and public health interventions related to depression |
| 23 | Du et al. The association between dietary quality, sleep duration, and depression symptoms in the general population: findings from cross-sectional NHANES study. BMC Public Health. 2024 Sep 27;24(1):2588. doi: 10.1186/s12889-024-20093-9. | Individuals affected by the individual and synergistic effect of an unhealthy diet and unhealthy sleep duration are more susceptible to experiencing depressive symptoms  Consistent link between inflammatory diets and depressive outcomes  Finding supports the hypothesis that avoiding pro-inflammatory foods and opting for anti-inflammatory diets may help prevent depression and its symptoms  The Healthy Eating Index (HEI) measures dietary quality and adherence to the Dietary Guidelines for Americans, representing an individual’s overall dietary patterns  Several cohort studies, including US, United Kingdom, and France, have shown that individuals with higher HEI scores have a lower risk of developing depressive symptoms |
| 24 | ElBarazi et al. Association between university student junk food consumption and mental health. Nutr Health. 2024 Dec;30(4):861-867. | Significant positive correlation between the consumption of ultra-processed, junk food and the increased risk of depression, anxiety, and stress, experienced by college students in Egypt |
| 25 | Ejtahed et al. Association between junk food consumption and mental health problems in adults: a systematic review and meta-analysis. BMC Psychiatry. 2024 Jun 12;24(1):438. | Consumption of junk food was associated with an increased of developing depression  Increased consumption of junk food has heightened the odds of depression and psychological stress in adult populations |
| 26 | Fabiano. Diet and depression. CMAJ. 2024 Oct 21;196(35):E1205-E1206. | Participants consuming a Mediterranean diet compared with controls reported moderate-to-large improvements in depressive symptoms |
| 27 | Fayyazi et al. Association between major dietary patterns and mental health problems among college students. J Educ Health Promot. 2024 Nov 29;13:440. | Strong inverse association between plant-based dietary pattern and depression |
| 28 | Ghanbarzadeh et al. Association of healthy eating index (2015) with depression and anxiety symptoms among Iranian adolescent girls. J Health Popul Nutr. 2024 Apr 2;43(1):44. doi: 10.1186/s41043-024-00529-z. | The Health Eating Index (leans towards being anti-inflammatory) is inversely correlated with depression and anxiety in Iranian adolescent girls  HEI was greater in the healthy participants than in those suffering from depression and anxiety |
| 29 | Gomes-da-Costa et al. Is a vegetarian diet beneficial for bipolar disorder? Relationship between dietary patterns, exercise and pharmacological treatments with metabolic syndrome and course of disease in bipolar disorder. Acta Psychiatr Scand. 2024 Oct;150(4):209-222. | Vegetarian diet pattern was associated with both, better clinical and metabolic parameters, and reduced depression scores in patients with bipolar disorder |
| 30 | Hepsomali et al. Dietary inflammation, sleep and mental health in the United Kingdom and Japan: A comparative cross-sectional study. Nutr Bull. 2024 Sep;49(3):396-407. | Compared with participants in Japan, those in the United Kingdom reported better overall mental wellbeing  Participants in Japan had better adherence to an anti-inflammatory diet and reported less severe depression, anxiety and stress and better subjective sleep quality, less sleep disturbances and daytime dysfunction, despite sleeping shorter |
| 31 | Hu et al. Identification of Chinese dietary patterns and their relationships with health outcomes: a systematic review and meta-analysis. Public Health Nutr. 2024 Oct 14;27(1):e209. | A ‘healthy Chinese diet’ characterized by whole grains, fresh vegetables, soyabean and products, fish and seafood, mushrooms and fungi, and nuts was associated with reduced risks of metabolic conditions, cognitive impairment and depressive symptoms, and better cardiovascular health  Animal-food diet was associated with a higher risk of depressive symptoms, as well as cardiovascular disease, diabetes, and abdominal obesity |
| 32 | Jones et al. Dietary factors associated with depressive symptoms in midlife women. Womens Health Rep (New Rochelle). 2024;5(1):997-1007. | To protect against depression, avoid high intake of polyunsaturated fats, omega-6 fatty acids, and sucrose; prioritize vitamin C and omega-3 e**icosapentaenoic acid**  Greater depression associated with higher intake of polyunsaturated fats, omega-6 linoleic acid, and sucrose but not associated with vitamin or mineral intake  Association between depressive symptoms and omega-6 linoleic acid while others reported either an inverse relationship or an association with the ratio of omega-6:omega-3; these fatty acids may modulate depression through anti-inflammatory processes  Findings support the Nurses’ Health Study findings for midlife women who completed a food frequency questionnaire every 4 years; a diet high in ultra-processed foods was a risk factor for depression   Limiting processed foods, which are typically high in sucrose and fat, may be effective in lowering brain-derived neurotrophic factor that plays a key role in the limbic system’s regulation of mood and depressive symptoms |
| 33 | Kamrani et al. Nourishing the mind: how the EAT-Lancet reference diet (ELD) and MIND diet impact stress, anxiety, and depression. BMC Psychiatry. 2024 Oct 19;24(1):709. doi: 10.1186/s12888-024-06165-5. | The ELD and MIND diet were both associated with reduced odds of depression |
| 34 | Kim et al. Fish consumption and depression in Korean population: The Korea National Health and Nutrition Examination Survey, 2013-2021. J Affect Disord. 2024 Aug 15;359:255-261. | High fish consumption is associated with lower risk of depression in Korean adults, especially in female adults |
| 35 | Kitabayashi et al. Relationship between food group-specific intake and depression. BMC Nutr. 2024 Jan 30;10(1):21. doi: 10.1186/s40795-024-00830-4. | Higher intake of eggs in men and vegetables in women may be linked to reduced depression  Odds of depression higher in both men and women with a low egg intake, and in women with a low vegetable intake |
| 36 | Lane et al. Sugar-sweetened beverages and adverse human health outcomes: An umbrella review of meta-analyses of observational studies. Annu Rev Nutr. 2024 Aug;44(1):383-404. | Convincing evidence (class I) supported direct associations between sugar-sweetened beverage consumption and risks of depression  These findings inform and provide support for population-based and public health strategies aimed at reducing sugary drink consumption for improved health |
| 37 | Leme et al. Overall diet quality, food groups and mental health disorders among Brazilians older than 15 years old: Brazilian National Health Survey - 2019. J Affect Disord. 2024 Jul 1;356:284-291. | Persons living with depression had significantly lower diet scores for vegetables/fruits, grains/roots, beans and higher scores for sweetened beverages, sweets and sugars, and high fat/sodium products  People with mental health disorders are more likely to have an unhealthy diet; relationships were slightly stronger with depression in particular food groups  Further studies are needed to help in the prevention of these disorders aided with healthy nutrition |
| 38 | Lin et al. Oxidative stress and inflammatory factors mediate the association between dietary antioxidant intake and depressive symptoms in middle and old-aged individuals: A cross-sectional study from NHANES. Gen Hosp Psychiatry. 2024;91:160-166. | Composite Dietary Antioxidant Index exhibits a significant negative association with depression, potentially mediated by oxidative stress and inflammation among middle-aged and older adults |
| 39 | Lin et al. Dietary flavonoid intake is associated with a lower risk of depressive symptoms in US adults: Data from NHANES 2007-2008, 2009-2010, and 2017-2018. J Affect Disord. 2024 Jan 15;345:293-299. | Moderate total flavonoids intake, but not high intake, was associated with lower odds of depressive symptoms suggesting a U-shaped association  Non-linear relationship was observed between total flavonoid intake and depressive symptoms |
| 40 | Lu et al. Adherence to the EAT-Lancet diet and incident depression and anxiety. Nat Commun. 2024 Jul 3;15(1):5599. | Greater adherence to the EAT-Lancet diet is associated with lower risks of incident depression, anxiety and their co-occurrence  This dietary pattern is primarily plant based and emphasizes the intake of vegetables, fruits, whole grains and nuts; it consists of moderate amounts of seafood and poultry and considerably limits the intake of red meat, added sugar and saturated fat  Adherence to the vegetable and fruit recommendation was associated with lower risks of depression  Adhering to the EAT-Lancet reference diet may serve as a promising modifiable target which enhances the primary prevention for co-occurrence of depression and anxiety |
| 41 | Lugon et al. Association between different diet quality scores and depression risk: the REGICOR population-based cohort study. Eur J Nutr. 2024 Dec;63(8):2885-2895. | Significant inverse association between diet quality and depression incidence was found in this population-based cohort study, independent of sociodemographic, health and lifestyle  Adherence to a healthy anti-inflammatory diet could be a complementary intervention for the prevention of depression |
| 42 | Marche et al. Is a plant-based diet effective to maintain a good psycho-affective status in old age? Results of a survey of a long-lived population from Sardinia. Nutr Neurosci. 2024;27(4):382-391. | Higher animal-derived protein consumption was reported to be beneficial for the affective status in an elderly population from Sardinia, whereas fresh fruit intake was associated with depressed mood, suggesting that a mere plant-based diet may prove to be inappropriate in the elderly living in traditional communities  Consumption of olive oil and lard was associated with decreased depression  A more balanced diet, including animal-derived foods, instead of an exclusive plant-dominant diet, may be more appropriate in the elderly, and abstention from animal-based food intake should not be recommended in advanced age to prevent depression |
| 43 | Marche et al. The impact of nutrition on psycho-affective status in an older Cretan population: a cross-sectional study. Eur J Nutr. 2024;63(6):2199-2207. | Among male participants, consumption of chicken meat, fish, cereals, cheese and fruit, was negatively associated with depression (i.e., protective), whereas among females, only vegetable consumption was associated with an increased risk of depression |
| 44 | Martins et al. Symptoms of depression and lifestyle in adolescents: A network analysis. Trends Psychiatry Psychother. 2024 Sep 25. doi: 10.47626/2237-6089-2024-0873. | Lifestyle domains with the highest expected influence were diet and nutrition (emphasis on anti-inflammatory food groups), over walking and stress management |
| 45 | Matison et al. Associations between fruit and vegetable intakes and incident depression in middle-aged and older adults from 10 diverse international longitudinal cohorts. J Affect Disord. 2024;359:373-381. | Beneficial association between higher fruit, but not vegetable, intake and incident depression, in analysis of adults 45+ years from 10 diverse international cohorts |
| 46 | Maurus et al. EPA guidance on lifestyle interventions for adults with severe mental illness: A meta-review of the evidence. Eur Psychiatry. 2024 Dec 10;67(1):e80. doi: 10.1192/j.eurpsy.2024.1766. | European Psychiatric Association guidelines include supports the application of lifestyle interventions that combine behavioral change techniques, dietary modification (consistent with heart health), and physical activity to reduce weight and improve cardiovascular health parameters in adults with severe mental illness including depression |
| 47 | Merino Del Portillo et al. Nutritional modulation of the gut-brain axis: A comprehensive review of dietary interventions in depression and anxiety management. Metabolites. 2024 Oct 14;14(10):549. doi: 10.3390/metabo14100549. | Direct relationship between what we eat and the state of our nervous system  Gut-brain axis is a complex system in which the intestinal microbiota communicates directly with our nervous system and provides it with neurotransmitters for its proper functioning  An imbalance in our microbiota due to poor nutrition will cause an inflammatory response that, if sustained over time and together with other factors, can lead to disorders such as anxiety and depression Changes in the functions of the microbiota-gut-brain axis have been linked to several mental disorders  It is believed that the modulation of the microbiome composition may be an effective strategy for a new treatment of these disorders |
| 48 | Naghshi et al. Association between different dietary carbohydrate and risk of depression, anxiety, and stress among female adolescents. Int J Prev Med. 2024 Dec 23;15:71. doi: 10.4103/ijpvm.ijpvm_291_23. | Association between dietary low-quality carbohydrate intake and mental disorders among female adolescent  Limiting the consumption of simple sugar may exert positive impacts on mental health |
| 49 | Qi et al. Association of dietary saturated fatty acid intake with depression: mediating effects of the dietary inflammation index. Front Nutr. 2024 Jun 14;11:1396029. doi: 10.3389/fnut.2024.1396029. | Dietary intake of saturated fatty acids is associated with the risk of depression in relation to the chain length of saturated fatty acids, and this may be due to the mediating effect of dietary inflammatory index |
| 50 | Qi et al. The effects of health risk behaviors to excess mortality in the population with depression: A cohort study based on NHANES data. J Affect Disord. 2024;356:233-238. | At least 1/5 of excess mortality for population with depression was attributable to health risk behaviors  Efforts should be made to address HRBs among population with depression  Unhealthy (pro-inflammatory) diet was determined based on the reported intake of fruits and vegetables in the 24 h before the survey examination; US Department of Agriculture food codes were used to identify fruits or vegetables from other foods  Single portion of fruit or vegetables was defined as that which weighs 30 g in dried forms or 80 g in non-dried forms; participants who consumed 3 portions while those portions spread out across less than three distinct fruits or vegetables were considered to have unhealthy diet |
| 51 | Qie et al. Combined healthy lifestyles and risk of depressive symptoms: A baseline survey in China. J Affect Disord. 2024;363:152-160. | Adherence to healthy lifestyle factors was associated with a reduced risk of having depressive symptoms among Chinese adults  Simple diet score was estimated according to the number of low-risk dietary habits: vegetable intake ≥300 g/day, fruit intake ≥200 g/day, red meat intake ≤500 g/wk, sugar-sweetened beverage consumption <1 can per day, tea intake ≥3x/wk; a diet score of 4-5 was considered healthy |
| 52 | Ross et al. Existing and future strategies to manipulate the gut microbiota with diet as a potential adjuvant treatment for psychiatric disorders. Biol Psychiatry. 2024;95(4):348-360. | Evidence strongly supports a diet high in plant-based foods, e.g., Mediterranean-type diet, improves mental health  Ascribed to the anti-inflammatory effects of certain microbial metabolites or a reduction of proinflammatory microbial products associated with plant-based nutrition |
| 53 | Saintila et al. Depression, anxiety, emotional eating, and body mass index among self-reported vegetarians and non-vegetarians: A cross-sectional study in Peruvian adults. Nutrients. 2024 May 29;16(11):1663. | Association between consuming a vegetarian diet and higher levels of anxiety and depressive symptoms |
| 54 | Sangsefidi et al. The association between dietary anti-oxidant quality score and psychological disorders among Iranian adults: a population-based study. Nutr Neurosci. 2024;27(1):12-19. | Depressive mood associated with the impaired antioxidant defense and inﬂammatory status, thus anti-oxidative eﬀects of these diets may explain the inverse association between dietary antioxidants intake with depression and anxiety  Average levels of dietary antioxidant quality score might be related to psychological distress but findings were inconclusive, that is, low score associated with higher psychological distress |
| 55 | Shakil et al. Comparative analysis of nutritional status among institutionalised and community-dwelling elderly women and its association with mental health status and cognitive function. J Family Med Prim Care. 2024;13(8):3078-3083. | Community-dwelling elderly were relatively healthy compared to old age home residents  Maintaining the nutritional, cognitive, and mental health of institutionalised and community-dwelling elderly women requires the implementation of physical and cognitive stimulation activities as well as interventions targeted at improving a healthy anti-inflammatory diet |
| 56 | Song et al. Association of macronutrient consumption quality, food source and timing with depression among US adults: A cross-sectional study. J Affect Disord. 2024;351:641-648. | Higher consumption of total carbohydrates increases the risk of depression, mainly driven by low-quality carbohydrates  High-quality carbohydrates can lower the risk of depression; reducing low-quality carbohydrates while increasing high-quality carbohydrates can lower the prevalence of depression  Negative correlation between the consumption of high-quality carbohydrates, including fruits, whole grains, and other red vegetables, and the risk of depression  Consumption of high-quality carbohydrates may be associated with reduced depression; replacing low-quality carbohydrates with high-quality carbohydrates throughout the day reduced risk of depression by 15%  Main source of low-quality carbohydrates is added sugar, which is positively correlated with the risk of depression  Total high-quality carbohydrate, total animal protein, and total vegetable protein were negatively associated with depression  Replacing low-quality carbohydrates with high-quality carbohydrates throughout the day reduced the risk of depression by approximately 15%  Consuming an appropriate amount of unsaturated fatty acids at breakfast can reduce the risk of depression, but a high-fat diet is not recommended |
| 57 | Soveid et al. Animal and plant protein intake association with mental health, tryptophan metabolites pathways, and gut microbiota in healthy women: a cross-sectional study. BMC Microbiol. 2024 Oct 7;24(1):390. doi: 10.1186/s12866-024-03534-8. | Significant inverse associations were found between a diet high in plant protein with mental disorders, while adhering to higher animal protein could predispose women to psychological stress |
| 58 | Sugden et al. What do climate change, nutrition, and the environment have to do with mental health? Am J Lifestyle Med. 2024 Sep 3:15598276241280245. doi: 10.1177/15598276241280245. | Whole plant-based diet proposed as a solution to both mental illness and climate change  Role of nutrition in gut microbiota and mental health, the impact diet has on greenhouse gases, the role of ultra-processed food, and environmental factors such as air pollution and increasing planetary heat and their growing impacts on mental health  Concluded that the promotion of plant-based foods has the potential to improve personal mental and physical health while improving planetary health |
| 59 | Tan et al. Higher caloric ratio of carbohydrate intake associated with increased risk of depression: A cross-sectional analysis of NHANES data from 2005 to 2020. J Affect Disord. 2024 Dec 1;366:59-65. | Higher calorie ratio of carbohydrate intake is significantly associated with an increased likelihood of depressive symptoms among U.S. adults |
| 60 | Tohidi et al. Comparison of Dutch healthy eating and healthy eating indexes and anthropometry in patients with major depression with health subjects: a case-control study. Front Nutr. 2024 May 6;11:1370562. doi: 10.3389/fnut.2024.1370562. | Healthy Eating Index 2015 and Dutch Healthy Eating Diet (both anti-inflammatory and high in anti-oxidants) have a significant relationship in reducing major depression |
| 61 | Ünal et al. Association of Mediterranean diet with sleep quality, depression, anxiety, stress, and body mass index in university students: A cross-sectional study. Nutr Health. 2024 Jan 27:2601060231207666. | University students showed poor adherence to the Mediterranean diet  Low adherence to the Mediterranean diet may have a negative impact on depression, stress, and sleep quality  Interventions to promote adherence to the Mediterranean diet may help improve university students' mental health |
| 62 | van Zonneveld, et al. An anti-inflammatory diet and its potential benefit for individuals with mental disorders and neurodegenerative diseases—a narrative review. Nutrients. 2024 Aug 10;16(16):2646. | Anti-inflammatory diet, like the BrAIN diet, rich in fruits, vegetables, whole grains, healthy fats, and fermented foods, shows promise as an adjunct in the management of mental disorders  Pro-inflammatory characteristics of meat may not support mental and brain health, suggesting that the consumption of meat should be minimized  Inclusion of fermented foods in the diet offers significant benefits for mental and brain health  Fermented foods contribute to gut health by promoting microbial diversity and reducing inflammation  These effects can support the gut–brain axis, potentially improving symptoms related to neurodegenerative diseases and mental health disorders  An anti-inflammatory diet can attenuate intestinal inflammation by modulating the gut microbiome; this diet comprises a higher plant than animal content and fermented dairy products, with a limited intake of processed meat and alcoholic and soft drinks |
| 63 | Vasmehjani et al. The associations between plant-based dietary indices with depression and quality of life and insomnia among Iranian adolescent girls in 2015. Sci Rep. 2024 May 22;14(1):11683. doi: 10.1038/s41598-024-61952-0. | An unhealthy plant-based dietary index was associated to an increased chance of depression  A healthy plant-based dietary index is associated with a lower odds of insomnia |
| 64 | Vega-Cabello et al. Plant-based diets and risk of multimorbidity: The Health and Retirement Study. J Nutr. 2024 Jul;154(7):2264-2272. doi: 10.1016/j.tjnut.2024.04.037. Epub 2024 May 4. | Higher adherence to the hPDI (healthful plant-based dietary index) was inversely associated with multimorbidity including depression among middle-aged and older adults  Plant-based diets that emphasize consumption of high-quality plant foods may help prevent the development of complex multimorbidity including depression |
| 65 | Wang et al. The mediating role of dietary inflammatory index on the association between eating breakfast and depression: Based on NHANES 2007-2018. J Affect Disord. 2024;348:1-7. | Pro-inflammatory diet (consistent with the standard western diet) and skipping breakfast were risk factors for depression  Consuming breakfast regularly to lower dietary inflammatory index and further reduce the risk of depression |
| 66 | Wang et al. Association of dietary overall antioxidant intake with all-cause and cause-specific mortality among adults with depression: evidence from NHANES 2005-2018. Food Funct. 2024 Apr 22;15(8):4603-4613. | Comprehensive dietary antioxidant intake may improve depressive symptoms and lower mortality risk among adults with depression  Overall dietary antioxidant intake was beneficially associated with all-cause and cancer mortality in depressed adults |
| 67 | Wang et al. Association between vegetable intake and major depressive disorder: results from National Health and Nutrition Examination Survey 2005-2018 and bidirectional two-sample Mendelian randomization. Public Health Nutr. 2024 Oct 24;27(1):e220. | Participants with low vegetable intake were associated with an increased risk of major mental disorders |
| 68 | Werneck et al. Adherence to the ultra-processed dietary pattern and risk of depressive outcomes: Findings from the NutriNet Brasil cohort study and an updated systematic review and meta-analysis. Clin Nutr. 2024 May;43(5):1190-1199. | Higher adherence to the ultra-processed dietary pattern was associated with a higher risk of developing depressive outcomes in the NutriNet Brasil cohort and in the meta-analysis |
| 69 | Yang et al. Association of dietary flavonoid intake with the prevalence and all-cause mortality of depressive symptoms: Findings from analysis of NHANES. J Affect Disord. 2024 Dec 1;366:44-58. doi: 10.1016/j.jad.2024.08.150. | Adequate intake of flavonoids, especially anthocyanidins and flavones (includes onions, kale, broccoli, apples, leaks, berries, celery, grapes, cabbage), is associated with reduced odds of depressive symptoms  Optimal intake ranges of flavonoid intake for mental health benefits were observed for all-cause mortality in population with depressive symptoms |
| 70 | Yildirim et al. The relationship of certain diseases and Dietary Inflammatory Index in older adults: A Narrative Review. Curr Nutr Rep. 2024 Dec;13(4):768-785. | Positive effects of anti-inflammatory nutrition on diseases including depression, contributing to clearer evidence of its protective effects on health |
| 71 | Zeng et al. Association of cardiovascular health using Life's Essential 8 with depression: Findings from NHANES 2007-2018. Gen Hosp Psychiatry. 2024;87:60-67 | Adhering to better cardiovascular health practices including heart healthy diet, estimated by the LE8 score, was correlated with lower odds of depression |
| 72 | Zhang et al. Association between dietary sugar intake and depression in US adults: a cross-sectional study using data from the National Health and Nutrition Examination Survey 2011-2018. BMC Psychiatry. 2024 Feb 8;24(1):110. | High-sugar diet can disrupt the gut microbiota, leading to depression  High dietary sugar intake in American adults is positively related to a higher prevalence of depression |
| 73 | Zhao et al. Association of dietary vitamin C intake with depression in adults: A cross-sectional study of NHANES from 2005 to 2020. J Affect Disord. 2024;358:113-120. | Negative association between dietary vitamin C intake and depression, as well as an L-shaped nonlinear relationship between vitamin C intake and depression  Healthy whole food, plant-based diets are high in vitamin C |

**Supplementary Table 2** Summary of recent 2024 literature related to reduced sedentarism and increased physical activity/physical exercise for potential modification of depression

**Reduced Sedentarism**

| No. | Study Title | Recommendations |
| --- | --- | --- |
| 1 | Guo et al. Association between long-term sedentary behavior and depressive symptoms in U.S. adults. Sci Rep. 2024 Mar 4;14(1):5247. doi: 10.1038/s41598-024-55898-6. | Long-term sedentary behavior may act as a risk factor for both depressive symptoms and moderate to severe depressive symptoms in American adults |
| 2 | Jiang et al. The relationship between sedentary behavior and depression in older adults: A systematic review and meta-analysis. J Affect Disord. 2024;362:723-730. | Total sedentary behavior in older adults is associated with an increased risk of depression  Mentally active sedentary behavior is associated with a decreased risk of depression  Passive sedentary behavior is not associated with the occurrence of depression |
| 3 | Li et al. Correlation between the physical activity volume and cognitive and mental capacity among older adult people in China: a cross-sectional study based on the 2020 CHARLS database. Front Public Health. 2024 Nov 20;12:1462570. doi: 10.3389/fpubh.2024.1462570. | Inactivity was associated with an increased risk of depression, cognitive impairment, and impaired daily physical functioning among older adult individuals in China |
| 4 | Liang et al. Associations of Reallocating Sedentary Time to Physical Activity and Sleep with Physical and Mental Health of Older Adults. Med Sci Sports Exerc. 2024;56(10):1935-1944. | Reallocating 30-min sedentary behavior to moderate-to-vigorous physical activity and sleep was associated with predicated improvements in all health outcomes, except blood pressure  Reallocating 30-min sedentary behavior to light physical activity resulted in predicted improvements in depressive symptoms as well as physical fitness and loneliness  For dose-effect relationships, reallocating 5-60 min of sedentary behavior to moderate-to-vigorous physical activity showed greatest benefits for all health outcomes |
| 5 | Park et al. Joint association of sedentary behavior and physical activity domains with depression in Korean adults: Cross-sectional study combining four biennial surveys (2016-2022). PLoS One. 2024 Oct 24;19(10):e0312029. doi: 10.1371/journal.pone.0312029. | Reducing sedentary behavior and encouraging leisure-time physical activity, and minimizing rigorous occupational physical activity may reduce depressive symptoms |
| 7 | Zhao et al. Research progress on the mechanism of exercise against depression.  World J Psychiatry. 2024;14(11):1611-1617. | Low physical activity and high sedentary behavior can significantly influence the onset of depression  On review of the literature, adolescents’ depressive symptoms suggest that every additional hour of sedentary behavior per day can cause an 8%-11% increase in depression scores by the age of 18.  When compared to those with lower levels, participants who maintained high or moderate sedentary behavior levels between the ages of 12 years and 16 years exhibited significantly higher depression scores by the age of 18  Research on older adults shows a close association between depression and sedentary behavior (e.g., watching television, using computers, and sitting in cars) and light-intensity physical activity (e.g., housework, gardening, and leisurely walks) |

**Physical Activity/Physical Exercise**

| No. | Study Title | Recommendations |
| --- | --- | --- |
| 1 | Alderman et al. Exercise for the prevention and treatment of depression. Curr Top Behav Neurosci. 2024;67:157-175. | Exercise and physical activity are lifestyle behaviors that can be used to prevent and treat depression  Engaging in regular physical activity is associated with a lowered risk of developing depression  Evidence for efficacy of exercise in treating depression, with effects that are comparable to those typically observed for antidepressant medications and psychotherapy  Aerobic and resistance exercise training results in moderate-to-large reductions in depressive symptoms, and exercise can be used as an adjunct to antidepressants and psychotherapy  American Psychiatric Association and National Institute of Mental Health, have not specifically endorsed the use of exercise as a treatment for depression, however statements from these organizations highlight the use of exercise as an ‘effective, cost-effective treatment for depression’ and that ‘exercise helps keep your psyche fit’ |
| 2 | Alghamdi et al. Prevalence of depression among subjects practicing aerobic vs. anaerobic exercise: a cross-sectional study. J Sports Med Phys Fitness. 2024;64(8):831-834. | All exercise may lead to individuals becoming less prone to depression; there is no advantage to undertaking aerobic exercise over anaerobic exercise  As long as there is good adherence and the appropriate number of sessions and amount of time, individuals should be encouraged to choose their type of exercise according to their needs and preferences |
| 3 | Alowaydhah et al. The effects of various types of physical exercise on health outcomes in older adults with depression: A systematic review and meta-analysis of controlled trials. Depress Anxiety. 2024 Jun 19;2024:9363464. | Multicomponent, aerobic, and tai chi forms of exercise appear most likely to reap benefits in depressed older adults; however, the type of benefit is determined by the type of exercise and should be considered when recommending a regime |
| 4 | Bendau et al. Exercise as treatment for “stress-related” mental disorders. Curr Neuropharmacol. 2024;22(3):420-436. | Most evidence targets unipolar depressive disorder and, secondly, anxiety disorders  Physical activity seems to be useful as a stand-alone-treatment as well as in combination with other psychotherapeutic or pharmacological treatments  Multiple intertwined physiological, psychological, and social mechanisms are assumed to mediate the beneficial effects. |
| 5 | Boschesi Barros et al. . Modifiable risk factors and excess mortality in depression: Data from the UK Biobank. Gen Hosp Psychiatry. 2024 Nov-Dec;91:11-17. doi: 10.1016/j.genhosppsych.2024.08.010. | All modifiable risk factors including low physical activity were associated with significantly increased age and sex–adjusted mortality hazard ratios |
| 6 | Bidzan-Wiącek et al. The relationship between physical activity and depressive symptoms in males: A systematic review and meta-analysis. Acta Psychol (Amst). 2024 Mar;243:104145. Doi: 10.1016/j.actpsy.2024.104145. | Men who engage in moderate physical activity present lower prevalence of depressive symptoms compared to reference subjects  Such associations were not found for low or high physical activity  Mental health benefits of physical activity could possibly be achieved at appropriate levels, at least in men |
| 7 | Bizzozero-Peroni et al. Daily Step Count and Depression in Adults: A Systematic Review and Meta-Analysis. JAMA Netw Open. 2024 Dec 2;7(12):e2451208. | Higher daily step counts were associated with fewer depressive symptoms in cross-sectional and longitudinal studies in the general adult population |
| 8 | Chen et al. The effects of aerobic exercise for depression: An umbrella review of systematic reviews and meta-analyses.  J Bodyw Mov Ther. 2024;40:2161-2172. | Aerobic exercise acts as an adaptive coping strategy that helps manage depression symptoms  Supervised aerobic exercises were effective for adults with depression in general  moderate levels of physical activity during leisure time reduce the risk of depression  Walking for more than 40 min and less than 20 min at an average pace is associated with a reduction in depression risk of 17% and 6%, respectively  Supervised aerobic exercise involving cycling or walking in either an individual or group setting is effective in treating depression in general adults when used either independently or in combination with other treatments such as antidepressants, exercise, or psychological therapy |
| 9 | Chen et al. Formulation of precise exercise intervention strategy for adolescent depression. Psych J. 2024 Apr;13(2):176-189. | Precision exercise intervention strategy (moderate-intensity aerobic exercise for 8-10 weeks, 3 times/week, 45-50 min/time) was constructed to improve adolescent depression |
| 10 | Chen et al. The effects of aerobic exercise for depression: An umbrella review of systematic reviews and meta-analyses. J Bodyw Mov Ther. 2024 Oct;40:2161-2172. | Substantial evidence that supports aerobic exercise to alleviate depressive symptoms  The positive synthesis of findings advocates for integrating aerobic exercise as a non-pharmacological treatment option |
| 11 | Correia et al. Analysis of the Effect of Different Physical Exercise Protocols on Depression in Adults: Systematic Review and Meta-analysis of Randomized Controlled Trials. Sports Health. 2024;16(2):285-294. | Physical exercise can be a way to reduce depression and used as a possible adjunctive tool for pharmacological and/or alternative treatments  Health professionals (e.g., exercise physiologists, physicians, nurses, psychologists) promote the practice of physical exercise as a complementary alternative and act early to prevent the worsening of depression |
| 12 | Cunha et al. Can resistance training improve mental health outcomes in older adults? A systematic review and meta-analysis of randomized controlled trials.  Psychiatry Res. 2024 Mar;333:115746. | Resistance training improved mental health outcomes (depressive and anxiety symptoms) in individuals with and without mental disorders, and some resistance training characteristics influenced the effect of resistance training on mental health; exercise prescriptions ranged on average 2-3x/wk 2-3 sets of 8-15 repetitions, with 1-2 min rest between; of 20 to 80% of repetition max; exercise types ranged from a single exercise to 9; duration of program ranged from 8 wks to 26 months  Prescription for best outcomes: 3x per week, 3 sets per exerciser, fewer exercises per session (≤ 6), and a shorter duration of the intervention (< 12 weeks) showed more efficiency in improving mental health parameters |
| 13 | Faronbi et al. Influence of physical activity on loneliness and depression among the older adults in Nigeria. Sci Rep. 2024 Nov 5;14(1):26781. Doi: 10.1038/s41598-024-77263-3. | Predictors of depression in this population are physical activity, age, loneliness, education, and low social engagement other than religious affiliation |
| 14 | Garcia-Estela et al. Evaluating the effect of exercise-based interventions on functioning in people with transdiagnostic depressive symptoms: A systematic review of randomised controlled trials. J Affect Disord. 2024;351:231-242. | Supervised exercise programs (8-12 wks, 3-4 sessions/wk) improve functioning in depressive patients  Including exercise as a routine add-on treatment for individuals with transdiagnostic depressive symptoms, including subthreshold cases, can improve functioning/quality of life and symptom severity  Should be considered standard clinical practice to recommend exercise  Effectiveness of exercise-based interventions on functioning in people with transdiagnostic depressive symptoms when compared with any other active control group or TAU depends on factors such as baseline functional status, intervention intensity, supervision during exercise, and delivery method (15 trials, 2064 participants)  Most significant improvement in functioning in people with transdiagnostic depressive symptoms, lasted 8–12 wks, with frequencies ranging from 3 h to four days/wk  Relatively little amounts oof physical activity can benefit mental health, with the greatest impact observed when transitioning from inactivity to any level of activity |
| 15 | Guimarães et al. Acute and chronic effects of physical exercise in inflammatory biomarkers in people with depression: A systematic review with meta-analysis. J Psychiatr Res. 2024 Nov;179:26-32. | People with depression have increased levels of pro-inflammatory cytokines compared to healthy subjects  Physical exercise can alleviate depressive symptoms and has anti-inflammatory properties |
| 16 | Guo et al. The triangular relationship of physical activity, depression, and inflammatory markers: A large cross-sectional analysis with NHANES data. J Affect Disord. 2024 Dec 15;367:589-597. | Positive link between depression and inflammatory marker, whereas PA was inversely correlated with both inflammatory marker and depression  Greatest reduction in the risk of depression when the level of PA was between 1200 and 1722 MET-min/wk (the optimal dosage of PA to minimize the likelihood of depression)  Inflammatory markers mediate the potential effects of physical inactivity on depression, ranging from 1.72 % to 6.25%  PA appear to protect against depression, in which inflammatory markers may play a mediating role |
| 17 | Gutierrez et al. Association between depressive symptoms, physical activity, and health factors in Hispanic emerging adults. Int J Environ Res Public Health. 2024 Jul 14;21(7):918. doi: 10.3390/ijerph21070918. | A threshold of physical activity, 600 MET min/wk, may confer protective effects against depressive symptoms |
| 18 | Herring et al. Resistance exercise for anxiety and depression: efficacy and plausible mechanisms. Trends Mol Med. 2024;30(3):204-206. | Resistance exercise produces significant anxiolytic and antidepressant effects in healthy and in clinical populations  There is substantial promise in evaluating the mechanisms underlying these effects of resistance muscle exercise due to the ability for future interventions and practice to target these mechanisms to maximize their effects and potentially optimize prescription via precision medicine approaches |
| 19 | Kawai et al. Association between physical activity duration and depressive symptoms in adolescents: A longitudinal study in a rural city in Japan. PLoS One. 2024 May 31;19(5):e0304783. doi: 10.1371/journal.pone.0304783. | In male adolescents with good mental health, a pattern of change from active to inactive in terms of physical activity due to lifestyle changes increased the risk of moving from no depressive tendency to increased depressive tendency  This association was weakened in females, suggesting that the influence of social support, such as communication with friends and family, on depressive symptoms was stronger in females than in males. |
| 20 | Kim et al. Impact of leisure physical activity and resistance exercise on the prevalence of depressive symptoms in Korean adults: Analysis of the Korean National Health and Nutrition Examination Survey. J Affect Disord. 2024 Jul 1;356:329-337. | Participation in leisure physical activity and resistance exercise was associated with a lower prevalence of depressive symptoms  Both leisure physical activity and resistance exercise should be encouraged as effective means for preventing depression |
| 21 | Kong et al. Effects of exercise interventions on physical, psychological and social outcomes in frail older adults: An overview of systematic reviews. J Clin Nurs. 2024 May 8. Doi: 10.1111/jocn.17214. | Physical activity at any level was the most effective intervention to reduce depression in frail elderly  Group-based exercise could provide intended activities and broaden social network, contributing to improve both frailty status and depression  Exercise interventions are key to improve physical, psychological and social outcomes in frail older adults |
| 22 | Kostick et al. Movement behaviors and mental health of Catholic priests in the eastern United States. J Relig Health. 2024 Jun;63(3):1867-1879. doi: 10.1007/s10943-023-01894-5. Epub 2023 Aug 17. | Priests who reported excessive recreational screentime (≥ 3 h/day) were more likely to have anxiety and depression, and who were physically inactive (< 150 min/wk) were more likely to have depression |
| 23 | Li et al. Physical activity and health-related quality of life in older adults: depression as a mediator. BMC Geriatr. 2024 Jan 5;24(1):26. Doi: 10.1186/s12877-023-04452-6. | Leisure-time, household, and work-related physical activity were negatively associated with depression, while positively affecting HRQoL in Chinese older adults  Relationships between different types and intensities of physical activity and HRQoL were mediated by depression  Interventions aimed at promoting purposeful exercise and different types of physical activity may have mental health benefits |
| 24 | Li et al. Optimal intensity and dose of exercise to improve university students’ mental health: A systematic review and network meta-analysis of 48 randomized controlled trials. Eur J Appl Physiol. 2024 Dec 18. Doi: 10.1007/s00421-024-05688-9. | Exercise improves symptoms of depression, anxiety, and stress in college students  WHO-recommended weekly dose of 600–1200 METs-min/wk during both very light and moderate exercise had a significant effect on reducing depression  A low threshold for intervening in depressive symptoms through exercise, only 150 METs-min per week  Very light, light, moderate, and vigorous exercise are all potentially effective exercise intensities for improving college students’ psychological well-being and that there were no significant differences in efficacy among the four exercise intensities  Vigorous exercise had the greatest effect on depression and stress |
| 25 | Li et al. Influence of aerobic exercise on depression in young people: a meta-analysis. BMC Psychiatry. 2024 Aug 20;24(1):571. | Evidence to support that the most effective exercise regimen, 40 min of moderate-intensity aerobic exercise 3x/wkk for 6 to 11 wks, showed more significant improvement in depression indicators in young people with depressive symptoms |
| 26 | Li et al. The relationship between mental health and physical activity among students from a private university: A cross-section study. Georgian Med News. 2024 Jul-Aug;(352-353):112-117. | Physical activity was significantly associated with mental health  It is necessary to take measures to reduce anxiety and depression in college students, to improve their understanding of the importance of a physically-active healthy lifestyle |
| 27 | Li et al. Influence of moderate-to-high intensity physical activity on depression levels: a study based on a health survey of Chinese university students. BMC Public Health. 2024 Apr 12;24(1):1023. doi: 10.1186/s12889-024-18433-w. | The lack of physical activity among Chinese college students is evident  Engaging in moderate to vigorous physical activity can reduce the likelihood of depression among college students  Moderate to vigorous physical activity achieves this reduction by enhancing college students' general self-efficacy and improving their physical health |
| 28 | Liu et al. The impact of single sessions of aerobic exercise at varying intensities on depressive symptoms in college students: evidence from resting-state EEG in the parietal region. BMC Psychiatry. 2024 Dec 18;24(1):928. Doi: 10.1186/s12888-024-06322-w. | Various intensities of a single bout of aerobic exercise can alleviate depressive symptoms in college students |
| 29 | Marinelli et al. Resistance training and combined resistance and aerobic training as a treatment of depression and anxiety symptoms in young people: A systematic review and meta-analysis. Early Interv Psychiatry. 2024 Aug;18(8):585-598. | Resistance training is an effective intervention in reducing depression and anxiety symptoms in young people, delivered across a range of settings. |
| 30 | Meyer et al. Feasibility and preliminary efficacy of a theory-informed resistance exercise training single-arm intervention for major depression. Psychol Sport Exerc. 2024 Jul;73:102642. | Resistance exercise training shows large anti-depressant effects in the treatment of multiple depressive disorder, thus is feasible and plausibly efficacious |
| 31 | Moss et al. The associations of physical activity and health-risk behaviors toward depressive symptoms among college students: Gender and obesity disparities. Int J Environ Res Public Health. 2024 Mar 26;21(4):401. Doi: 10.3390/ijerph21040401. | Enhancing mental health by improving physical activity and eliminating health-related behavior risks should be tailored toward at-ris  k demographics |
| 32 | Noetel et al. Effect of exercise for depression: Systematic review and network meta-analysis of randomised controlled trials. BMJ. 2024 Feb 14;384:e075847. | Various exercise modalities are effective (walking, jogging, mixed aerobic exercise, strength training, yoga, tai chi, qigong) and well tolerated (especially strength training and yoga)  Effects appeared proportional to the intensity of exercise prescribed and were stronger for group exercise and interventions with clear prescriptions  Preliminary evidence suggests interactions between types of exercise and patients’ personal characteristics |
| 33 | Rahmati et al. Physical activity and prevention of mental health complications: An umbrella review. Neurosci Biobehav Rev. 2024 May;160:105641. | Study provides further evidence for the protective effects of physical activity against depression and anxiety or stress-related disorders  Association between higher levels of physical activity and a reduced risk of depression was consistent across studies consistent with a significant reduction in the subsequent risk of incident depression  Higher levels of physical activity are associated with reduced risk of depressive symptoms or major depressive disorder regardless of age (adults and older adults), sex (males and females), and region (North America, Europe, Asia, and Australia)  Protective effects were observed for low and moderate-intensity physical activity, but not for high-intensity physical activity |
| 34 | Rossi et al. Strength training has antidepressant effects in people with depression or depressive symptoms but no other severe diseases: A systematic review with meta-analysis. Psychiatry Res. 2024 Apr;334:115805. | Duration of the intervention in weeks, weekly frequency of the intervention, number of sets and number of repetitions can influence the antidepressant effects  These variables have a small role on the variation of the effect  Strength training has a moderate antidepressant effect  Strength training should be considered an adjunct strategy to treat depression or depressive symptoms |
| 35 | Su et al. Expectations regarding school decreases emotional distress among college students in Western China: the buffering role of physical exercises. Front Public Health. 2024 Nov 6;12:1412199. doi: 10.3389/fpubh.2024.1412199. | Expectations regarding school and school belongingness, particularly the exclusion component, emerge as pivotal factors influencing emotional distress among college students in Western China  Physical exercise is a promising targeted intervention for alleviating emotional distress within this demographic |
| 36 | Soini et al. Physical activity and specific symptoms of depression: A pooled analysis of six cohort studies. J Affect Disord. 2024 Mar 1;348:44-53. Doi: 10.1016/j.jad.2023.12.039. | Low physical activity may be linked to depressive symptoms particularly through the symptoms of anhedonia and fatigue  Their association with physical activity amount follows a dose-response pattern and is independent of activity intensity |
| 37 | Tang et al. Optimal dose and type of exercise to improve depressive symptoms in older adults: A systematic review and network meta-analysis. BMC Geriatr. 2024 Jun 7;24(1):505. | U-shaped relationship between exercise and the alleviation of depressive symptoms in the older adults, and our study provided statistically (350–1000 METs-min/wk) and clinically (600–970 METs-min/wk) meaningful dosage ranges with the optimal dose (800 METs-min/wk)  Beneficial effects of moderate exercise dosages on the mitigation of depressive symptoms in tolder adults may originate from physiological and psychological mechanisms  The relationship between exercise dosage and depressive symptoms in the elderly varied depending on the type of exercise. i.e., AE, qigong, tai chi, walking, and yoga exhibited a non-linear increase in their efficacy in alleviating depressive symptoms with increasing exercise dosage  Dosage-response relationship for resistance training in mitigating depressive symptoms resembled the overall pattern of exercise (U-shaped relationship)  Comparing exercise dosages across exercise types, only resistance training had an exercise dosage exceeding 1000 METs*min/wk, unlike the other exercise types  Heart rate variability decreased with the severity of depressive symptoms  Resistance training does not appear to have the same consistent efficacy in improving heart rate variability as aerobic exercise  AE, rather than walking, demonstrated its optimal efficacy in alleviating depressive symptoms in older adults at a dosage of 1000 METs*min/wk  Walking was effective in alleviating depressive symptoms in older adults at dosages exceeding 250 METs*min/wk  AE (≥ 820 METs-min/wk), resistance training (520–1000 METs-min/wk), Walking (≥ 650 METs-min/wk), and yoga (≥ 680 METs-min/wk) all exhibited clinical benefits within their respective dosage ranges  Various exercises, including walking, AE, yoga, qi gong, resistance training, and tai chi, effectively alleviate depressive symptoms in older adults  Early initiation of exercise is beneficial, but its efficacy diminishes from the age of 80, and beyond 81, exercise no longer significantly alleviates depressive symptoms  **AE= aerobic exercise* |
| 38 | Tao et al. Effects of high-intensity interval training on depressive symptoms: A systematic review and meta-analysis. J Psychosom Res. 2024 May;180:111652. Doi: 10.1016/j.jpsychores.2024.111652. | High intensity interval training confers benefits in mitigating depressive symptoms.  Compared to non-active control group, high intensity interval training yields moderate improvements in depressive symptoms |
| 39 | Tebar et al. Association of meeting 24-hour movement guidelines with anxiety and depressive symptoms in adults. BMC Public Health. 2024 Dec 18;24(1):3509. doi: 10.1186/s12889-024-21038-y. | Intervention strategies for reducing anxiety and depressive symptoms should target the meeting of more than one movement guidelines, such as increasing physical activity, reducing sedentary time, and promoting adequate sleep time for adults. |
| 40 | Tian et al. Comparative efficacy of various exercise types and doses for depression in older adults: A systematic review of paired, network and dose–response meta-analyses. Age Ageing. 2024 Oct 1;53(10):afae211. | U-shaped relationship between exercise dose and its impact on depression in older adults  Deviating from this range may reduce the maximum potential benefits  Interventions exceeding 1000 METs-min/wk did not demonstrate substantial advantages, suggesting that increasing the volume of exercise does not always lead to greater improvements  A recent meta-analysis has shown that there is a direct correlation between the increase in weekly energy expenditure and the improvement in participants’ depression levels  Excessive exercise, by increasing physical stress and fatigue, can lead to worsening mental health problems in older people, particularly an increased risk of anxiety and depression  Specific types of exercise, such as RE at around 560 METs-min/wk, can result in clinical improvements even when the dose is below the recommended minimum of 600 METs-min/wk  AE, RE, ME and MBE interventions reduce depression in older adults  RE and MBE were more effective than AE and ME in reducing depression in older adults  It took ∼140 min/wk for RE and 100 min/wk for ME to reduce depression in older adults  **AE= aerobic exercise, RE = resistance exercise. ME= mixed exercise, MBE = mind-body exercise* |
| 41 | Tian et al. Optimal exercise modality and dose to improve depressive symptoms in adults with major depressive disorder: A systematic review and Bayesian model-based network meta-analysis of RCTs.  J Psychiatr Res. 2024;176:384-392. | A nonlinear U-shaped dose-response relationship was observed between exercise and depression levels  Findings corroborated the efficacy of physical activity interventions in ameliorating depressive symptoms among individuals afflicted by major depressive disorder  Exercise, regardless of its type reduced depression after a minimum intervention period of four wks  Aerobic and mind-body exercises were more effective  Minimal effective dose was estimated at 320 METs-min/wk and the optimal response at 860 METs-min/wk  Our review highlighted different dose-response profiles for various physical activity interventions  Optimal dose of mind-body exercise is lower than that of other positive intervention modalities; optimal response at 640 METs-min/wk  Significant benefits of aerobic, resistance, mixed, and mind-body exercise interventions in improving clinical depressive symptoms in patients with major depressive disorders  Aerobic exercise required 140 min/wk to improve depressive symptoms  Mind-body exercise required only 50 min/wk to produce similar effects |
| 42 | Veronese et al. Physical activity and persistence of supra-threshold depressive symptoms in older adults: A ten-year cohort study. Psychiatry Res. 2024 Dec;342:116259. doi: 10.1016/j.psychres.2024.116259. | Among adults with depression, higher levels of physical activity were associated with a reduced persistence of depression  These real-world data complement evidence on efficacy of exercise as a treatment for depression and can inform clinical guidelines |
| 43 | Wang et al. Effects of physical activity and depressive symptoms on cognitive function in older adults: National Health and Nutrition Examination Survey. Neurol Sci. 2024 Jan;45(1):299-308. doi: 10.1007/s10072-023-07250-5. | High levels of physical activity were independently associated with a lower incident cognitive impairment  The severity of depression was positively correlated with an increased risk of cognitive impairment |
| 44 | Wei et al. The effect of physical activity on depression in university students: The mediating role of self-esteem and positive psychological capital. Front Psychol. 2024 Sep 24;15:1485641. | Notable association among physical exercise, self-esteem, positive psychological capital, and depression among college students  Physical exercise has a negative impact on predicting depressive moods in college students |
| 45 | Weng et al. Effects of physical training on depression and related quality of life in pre-frail and frail older adults: a systematic review and meta-analysis. J Nutr Health Aging. 2024 Jun;28(6):100237. | Positive effects of physical training on depression and related quality of life were evident for people with frailty  No positive results were observed in pre-frail older adults, indicating the need for further investigation in this subgroup |
| 46 | Xu et al. Associations between regular physical exercise and physical, emotional, and cognitive health of older adults in China: an 8-year longitudinal study with propensity score matching. Front Public Health. 2024 Apr 9;12:1301067. Doi: 10.3389/fpubh.2024.1301067. | Engaging in long-term structured and repetitive physical exercise can have a significant positive effect on reducing depressive symptoms and improving the physical function of older adults  Incorporating regular physical exercise into the lifestyle of older adults is an effective strategy for promoting healthy aging |
| 47 | Xu et al. Association of physical activity and sedentary behaviour with depressive symptoms in Chinese adults. J Sports Sci. 2024 Dec;42(23):2191-2198. doi: 10.1080/02640414.2024.2425909. | High level of physical activity may not offset the increased risk of depressive symptoms associated with high amounts of sedentary behavior  These behaviours should be considered jointly to obtain optimal prevention effects |
| 48 | Xu et al. The effect of walking on depressive and anxiety symptoms: Systematic review and meta-analysis. JMIR Public Health Surveill. 2024 Jul 23;10:e48355. | Walking can be adopted as an evidence-based intervention for reducing depression and anxiety  Various forms of walking can be effective in reducing symptoms of depression and anxiety, and the effects of walking are comparable to active controls |
| 49 | Ye et al. The effect of physical activity on depression: a lagged mediation study of school burnout. BMC Public Health. 2024 Dec 18;24(1):3491. Doi: 10.1186/s12889-024-21003-9. | Only high-intensity physical activity was more effective in reducing depression and school burnout |
| 50 | Yuping et al. The optimal type and dose of exercise for elevating brain-derived neurotrophic factor levels in patients with depression: A systematic review with pairwise, network, and dose-response meta-analyses. Depress Anxiety. 2024 Dec 21;2024:5716755. | Combined aerobic and resistance exercise, resistance exercise, and yoga are effective interventions for enhancing bran-derived neurotropic factor levels in patients with depression, with Qigong, mindfulness, and continuous aerobic exercise being comparatively less effective.  A positive nonlinear dose-response relationship between exercise volume and bran-derived neurotropic factor levels was observed |
| 51 | Zhang et al. The effect of physical exercise on depression among college students: A systematic review and meta-analysis. PeerJ. 2024 Sep 23;12:e18111. | Integrating suitable physical exercise into the routines of individuals experiencing depressive symptoms can be a means of substantially alleviating depression and enhancing both physical and mental well-being  Physical exercise recommendation for college students is to engage between 30 to 60 min, more than or equal 3 sessions/wk, and physical exercise sessions last longer than 12 wks to develop a long-term habit of regular physical exercise |
| 52 | Zhang et al. Cross-sectional association between 24-hour movement guidelines and depressive symptoms in Chinese university students. PeerJ. 2024 Apr 15;12:e17217. doi: 10.7717/peerj.17217. | Prevalence of meeting the 24-h movement guidelines in Chinese university students was relatively low and should be enhanced through multiple strategies  Meeting the 24-h movement guidelines was associated with lower risk for depression in Chinese young adults  Moving more, sitting less and sleeping well in this population may reduce the occurrence of depression |
| 53 | Zhao et al. Research progress on the mechanism of exercise against depression.  World J Psychiatry. 2024;14(11):1611-1617. | Exercise is an effective alternative treatment option because of antidepressant drugs limited acute and long-term efficacy as well as the frequent treatment resistance and side effects in patients  Acute exercise transiently modulates circulating serotonin, norepinephrine and brain-derived neurotrophic factor levels as well as various immune-inflammatory mechanisms in depression’s clinical cohorts  Exercise activates the hypothalamus-pituitary axis, increases cortisone levels, prevents/reverses depression, improves cognition, and promotes plasticity of the brain structure and function  2.5 h of brisk walking/wk is the minimum physical activity level for combating depression  Yoga, strength training, and walking/jogging have demonstrated the strongest antidepressant effects  Less low-intensity activity can exert a positive impact on depression  Exercise is an effective alternative therapy for depression and can be included in clinical treatment plans |
| 54 | Zhao et al. The influence of lifestyle habits on levels of depression among rural middle school students in Northeastern China. Front Public Health. 2024 Jan 29;12:1293445. | Among middle school students in rural Northeast China, the depression level of females was significantly higher than that of males  Poor quality sleep, low levels of physical activity, low household income, and long screen time were positively associated with depression |
| 55 | Zhou et al. The effect of replacing sedentary behavior with different intensities of physical activity on depression and anxiety in Chinese university students: an isotemporal substitution model. BMC Public Health. 2024 May 23;24(1):1388. doi: 10.1186/s12889-024-18914-y. | Replacing 30 min of sedentary behavior in daily life with moderate-vigorous physical activity may alleviate depression and anxiety symptoms in university students |
